# Promoting Proton Donation through Hydrogen Bond Breaking on Carbon Nitride for Enhanced H_2_O_2_ Photosynthesis

**DOI:** 10.1021/acsnano.4c04797

**Published:** 2024-07-26

**Authors:** Yao Lu, Yanzhen Guo, Shao Zhang, Lejing Li, Ruibin Jiang, Dieqing Zhang, Jimmy C. Yu, Jianfang Wang

**Affiliations:** † Department of Physics, The Chinese University of Hong Kong, Shatin, Hong Kong SAR 999077, China; ‡ Henan Provincial Key Laboratory of Nanocomposites and Applications, Institute of Nanostructured Functional Materials, Huanghe Science and Technology College, Zhengzhou, Henan 450006, China; § The Education Ministry Key Lab of Resource Chemistry, Joint International Research Laboratory of Resource Chemistry, Ministry of Education, and Shanghai Key Laboratory of Rare Earth Functional Materials, College of Chemistry and Materials Science, Shanghai Normal University, Shanghai 200234, China; ∥ Department of Chemistry, The Chinese University of Hong Kong, Shatin, Hong Kong SAR 999077, China; ⊥ School of Materials Science and Engineering, Shaanxi Normal University, Xi’an, Shaanxi 710119, China

**Keywords:** graphitic carbon nitride, hydrogen bonds, nitrogen vacancies, oxygen reduction reaction, photocatalytic H_2_O_2_ production, water oxidation reaction

## Abstract

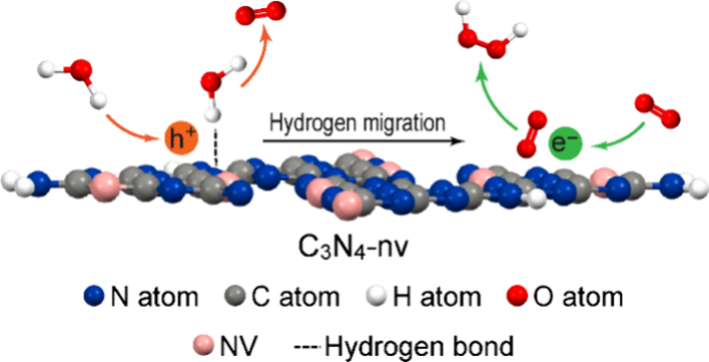

Photocatalytic H_2_O_2_ production has attracted much attention as an alternative way to the industrial anthraquinone oxidation process but is limited by the weak interaction between the catalysts and reactants as well as inefficient proton transfer. Herein, we report on a hydrogen-bond-broken strategy in carbon nitride for the enhancement of H_2_O_2_ photosynthesis without any sacrificial agent. The H_2_O_2_ photosynthesis is promoted by the hydrogen bond formation between the exposed N atoms on hydrogen-bond-broken carbon nitride and H_2_O molecules, which enhances proton-coupled electron transfer and therefore the photocatalytic activity. The exposed N atoms serve as proton buffering sites for the proton transfer from H_2_O molecules to carbon nitride. The H_2_O_2_ photosynthesis is also enhanced through the enhanced adsorption and reduction of O_2_ gas toward H_2_O_2_ on hydrogen-bond-broken carbon nitride because of the formation of nitrogen vacancies (NVs) and cyano groups after the intralayer hydrogen bond breaking on carbon nitride. A high light-to-chemical conversion efficiency (LCCE) value of 3.85% is achieved. O_2_ and H_2_O molecules are found to undergo a one-step two-electron reduction pathway by photogenerated hot electrons and a four-electron oxidation process to produce O_2_ gas, respectively. Density functional theory (DFT) calculations validate the O_2_ adsorption and reaction pathways. This study elucidates the significance of the hydrogen bond formation between the catalyst and reactants, which greatly increases the proton tunneling dynamics.

H_2_O_2_ is an indispensable chemical and an eco-friendly oxidant that finds extensive applications in chemical synthesis, environmental treatment, medical disinfection, and the pulp and paper industry.^1−5^ The industrial production of H_2_O_2_ is predominantly achieved by the anthraquinone cycle process.^6^ The process involves four steps, which are hydrogenation of anthraquinone in an organic solvent, oxidation of the hydrogenized anthraquinone in an O_2_-rich environment, extraction of the generated H_2_O_2_ and recycling of anthraquinone, and purification and concentration of H_2_O_2_.^7^ H_2_O_2_ is produced in the oxidation process of the hydrogenized anthraquinone. This industrial process is capable of large-scale production. Nevertheless, this multistep process consumes a large amount of energy. The hydrogen gas utilized in the hydrogenation of anthraquinone is difficult to handle and store. The generation of byproducts from anthraquinone and hydrogenized anthraquinone necessitates the use of a large amount of organic solvents, resulting in waste generation.

Various alternative approaches have been developed to overcome the challenges in the current industrial production process. Among these approaches, light-driven H_2_O_2_ synthesis has been emerging as one of the most appealing methods for H_2_O_2_ production.^8,9^ Photocatalytic H_2_O_2_ production typically includes several fundamental steps: light harvesting; generation, transfer, and separation of photogenerated electron–hole pairs; O_2_ and/or H_2_O adsorption; surface redox reactions; and H_2_O_2_ desorption.^10^ Notably, this process relies exclusively on four key components: catalysts, O_2_, H_2_O, and solar energy. In addition, sacrificial agents are required for most reported catalysts.^11^ Two-electron oxygen reduction reaction (ORR) and two-electron water oxidation reaction (WOR) represent two promising pathways for achieving photocatalytic H_2_O_2_ production.^12−14^ Among these pathways, the two-electron ORR has mainly been investigated for H_2_O_2_ production due to its superior thermodynamic favorability compared to the two-electron WOR. The ORR process can be classified into direct one-step two-electron reduction (eq 1) and indirect two-step single-electron reduction (eqs 2 and 3). The thermodynamics for H_2_O_2_ shows that the reaction potential of the one-step two-electron ORR is more positive than that of the two-step one-electron pathway. The direct ORR is thus thermodynamically favorable. In contrast, the direct ORR is less kinetically favorable because of two-electron transfer compared with the indirect pathway, which is only one-electron transfer for each step. For the indirect ORR, the reduction of O_2_ to superoxide radical (•O_2_^–^) is the rate-determining step since its reaction potential is more negative. The generated intermediate •O_2_^–^ might react with organic compounds in the reaction system or be reoxidized by hot holes to produce singlet ^1^O_2_ (eq 4). This is the competitive reaction to the H_2_O_2_ generation from the reduction of the intermediate •O_2_^–^.
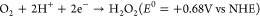
1
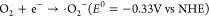
2
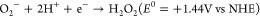
3
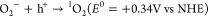
4However, the photocatalytic H_2_O_2_ production is limited by the weak interaction between the catalyst and O_2_ gas as well as inefficient proton-coupled electron transfer. In most protonation processes, H_2_O molecules are utilized as the proton source. Proton donation is restricted by the sluggish reaction kinetics in the oxidation of H_2_O molecules.^15^ In addition, the protons are easier to combine into H_2_ gas, which further limits the H_2_O_2_ production efficiency.

Photocatalysts employed for H_2_O_2_ production can be broadly classified into three types, namely, metal oxides,^16^ organic materials,^17−19^ and carbon nitrides.^20,21^ Among these types of photocatalysts, g-C_3_N_4_ has emerged as a widely employed photocatalyst for photocatalytic H_2_O_2_ production owing to its advantageous features, including cost-effectiveness, facile synthesis, appropriate band gap structure, thermal and chemical stability, and environmentally friendly nature.^22−24^ g-C_3_N_4_ possesses a graphitic stacking structure of C_3_N_4_ layers consisting of tri-*s*-triazine units connected through planar amino groups.^25−27^ The photocatalytic efficiency of g-C_3_N_4_ in H_2_O_2_ production has been demonstrated,^28^ wherein the formation of 1,4-endoperoxide species on the g-C_3_N_4_ surface suppresses the undesired one-electron ORR to •OOH but preferentially accelerates the selective two-electron ORR to H_2_O_2_.^25,29^ The selectivity of two-electron ORR toward H_2_O_2_ production over g-C_3_N_4_ is therefore high. As a typical layered material, g-C_3_N_4_ exhibits strong intralayer chemical bonding and weak interlayer van der Waals interactions (Figure 1a,b),^30^ which has been characterized to be shown as two diffraction peaks at 13.1 and 27.2° in the X-ray diffraction (XRD) pattern (Figure 1c), respectively.^31^ Nevertheless, despite the dominance of covalent bonding within the intralayer framework, abundant hydrogen bonds between the NH/NH_2_ groups at the edge of the melon strands exist owing to the incomplete polymerization of the nitrogen-containing precursors.^32,33^ These hydrogen bonds serve as binders to interconnect the strands of the melon units and maintain the long-range atomic-order pattern within the intralayer framework. However, the existence of intralayer hydrogen bonds on carbon nitride also leads to the deficiency of exposed N atoms, which hinders the hydrogen bond formation between exposed N atoms on carbon nitride and H_2_O molecules. N atoms on carbon nitride are supposed to be proton buffering sites. The deficiency of exposed N atoms therefore hinders the proton transfer from adsorbed H_2_O molecules to carbon nitride. Moreover, the presence of hydrogen bonds makes charge carrier transport difficult within the intralayer framework of g-C_3_N_4_ because of a large electrostatic potential barrier of 7.9 eV across the intralayer hydrogen-bond-located regions, thereby impeding intralayer charge carrier transport and therefore photocatalytic activities.^34^ Therefore, breaking the intralayer hydrogen bonds in g-C_3_N_4_ plays an important role in the enhancement of the photocatalytic activity.

**Figure 1:**
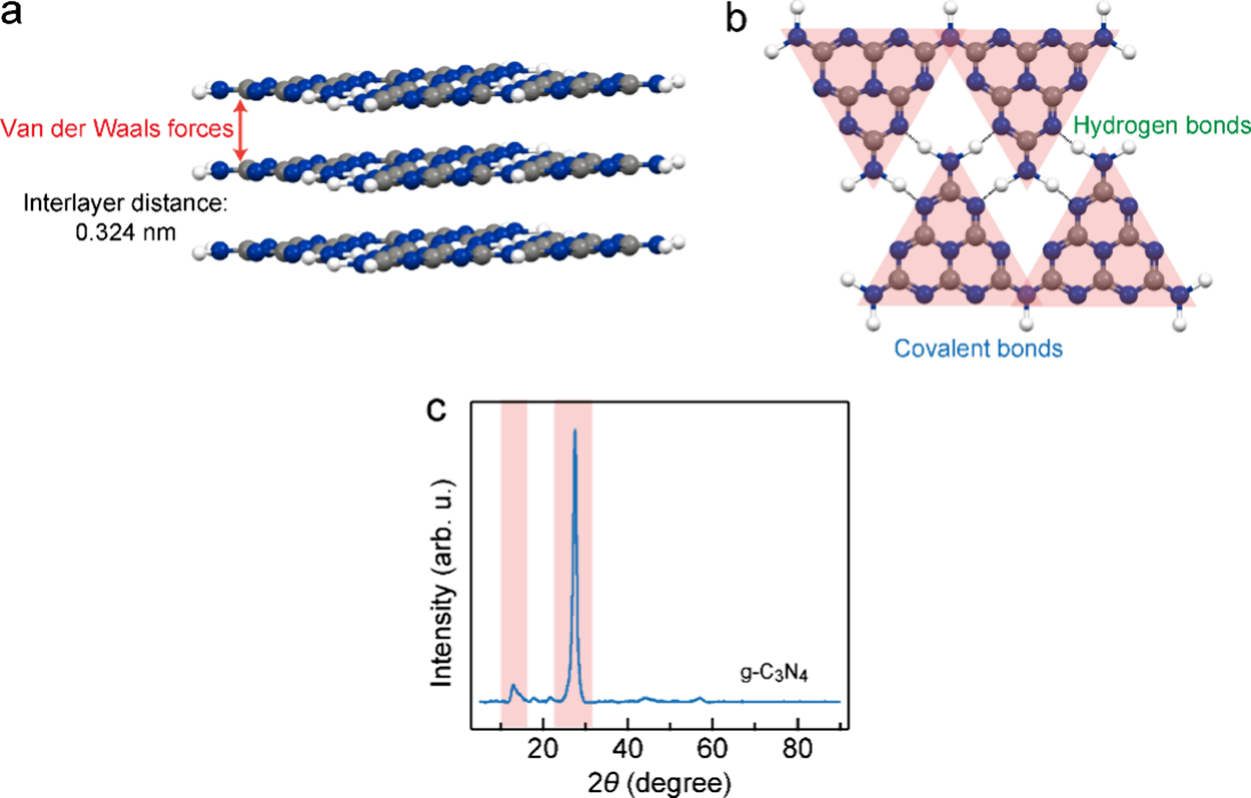
Atomic structure of layered g-C_3_N_4_ with intralayer hydrogen bonds. (a) Side view. (b) Top view. The C, N, and H atoms are represented by the dark-gray, dark-blue, and white balls, respectively. The melon strands are marked by the light pink triangles. (c) XRD pattern of g-C_3_N_4_.

The importance of the hydrogen bond formation between catalysts and reactants in catalysis has been demonstrated in several intriguing studies. For instance, hydrogen bond interactions in nitrogen photofixation have been found to enhance N_2_ activation. Notably, S atom-modified porous Cu catalysts exhibit high catalytic activities, where the formed S–H bonds facilitate N_2_ activation through hydrogen bonding.^35^ Moreover, the proton activity within the interfacial layer is of vital importance to proton-coupled electron transfer kinetics. Modification of the local proton activity with various protic ionic liquids in the interfacial layer of Au and Pt has been shown to substantially enhance the oxygen reduction activity, exhibiting a volcano relationship with the p*K*_a_ of the ionic liquid. This enhancement is ascribed to the favorable proton-coupled electron transfer (proton tunneling) kinetics facilitated by enhanced hydrogen bonding between the ORR products and the ionic liquid.^36^ Hydrogen bonds, both on the catalyst surface and in the bulk solution, offer valuable opportunities for tailoring the reaction pathway for selective photocatalysis. In the photocatalytic dehydrocoupling of ethanol over the Au/CdS catalyst, the presence of hydrogen bonds inhibits the oxidation and reverse reaction of α-hydroxyethyl radicals. Manipulating hydrogen bonds through water addition promotes the hydrogen bond formation between adsorbed α-hydroxyethyl radicals and H_2_O molecules, strengthens the hydrogen bonding between α-hydroxyethyl radicals and ethanol, and thus facilitates the desorption of α-hydroxyethyl radicals from the catalyst surface.^37^ Considering the crucial role of hydrogen bonds in catalysis, investigating their effect on H_2_O_2_ photosynthesis over carbon nitride becomes highly desirable. The enhancement of hydrogen bonding between the catalyst surface and reactants holds promise for enhancing photocatalytic H_2_O_2_ production.

In this work, the interaction between the catalyst surface and reactants during H_2_O_2_ photosynthesis is enhanced through the intralayer hydrogen bond breaking in carbon nitride. The breaking of the intralayer hydrogen bonds in carbon nitride is realized through post-thermal treatment in an inert atmosphere. The hydrogen-bond-broken carbon nitride photocatalyst exhibits an extensively enhanced H_2_O_2_ photosynthesis efficiency. The reaction mechanism studies show that O_2_ molecules are reduced to H_2_O_2_ by photoinduced electrons through a one-step two-electron reduction pathway, while H_2_O molecules are oxidized to O_2_ molecules by photoinduced holes through a four-electron oxidation pathway. Importantly, the decomposition of H_2_O_2_ is mitigated in the hydrogen-bond-broken carbon nitride. The study shows that the hydrogen bond breaking in carbon nitride leads to the formation of NVs and cyano (−C≡N) groups, which are both active sites for the adsorption and reduction of O_2_ gas toward H_2_O_2_ photosynthesis. The hydrogen-bond-broken carbon nitride is also found to have more exposed N atoms, which can form hydrogen bonds with H_2_O molecules. The formation of hydrogen bonds between exposed N atoms in the hydrogen-bond-broken carbon nitride and H_2_O molecules greatly contribute to the enhancement of proton-coupled electron transfer from the adsorbed H_2_O molecules to carbon nitride. In other words, the exposed N atoms in hydrogen-bond-broken carbon nitride provide proton buffering sites, which promotes the proton donation.

## Results and Discussion

### Photocatalyst Synthesis and Characterization

**Figure 2:**
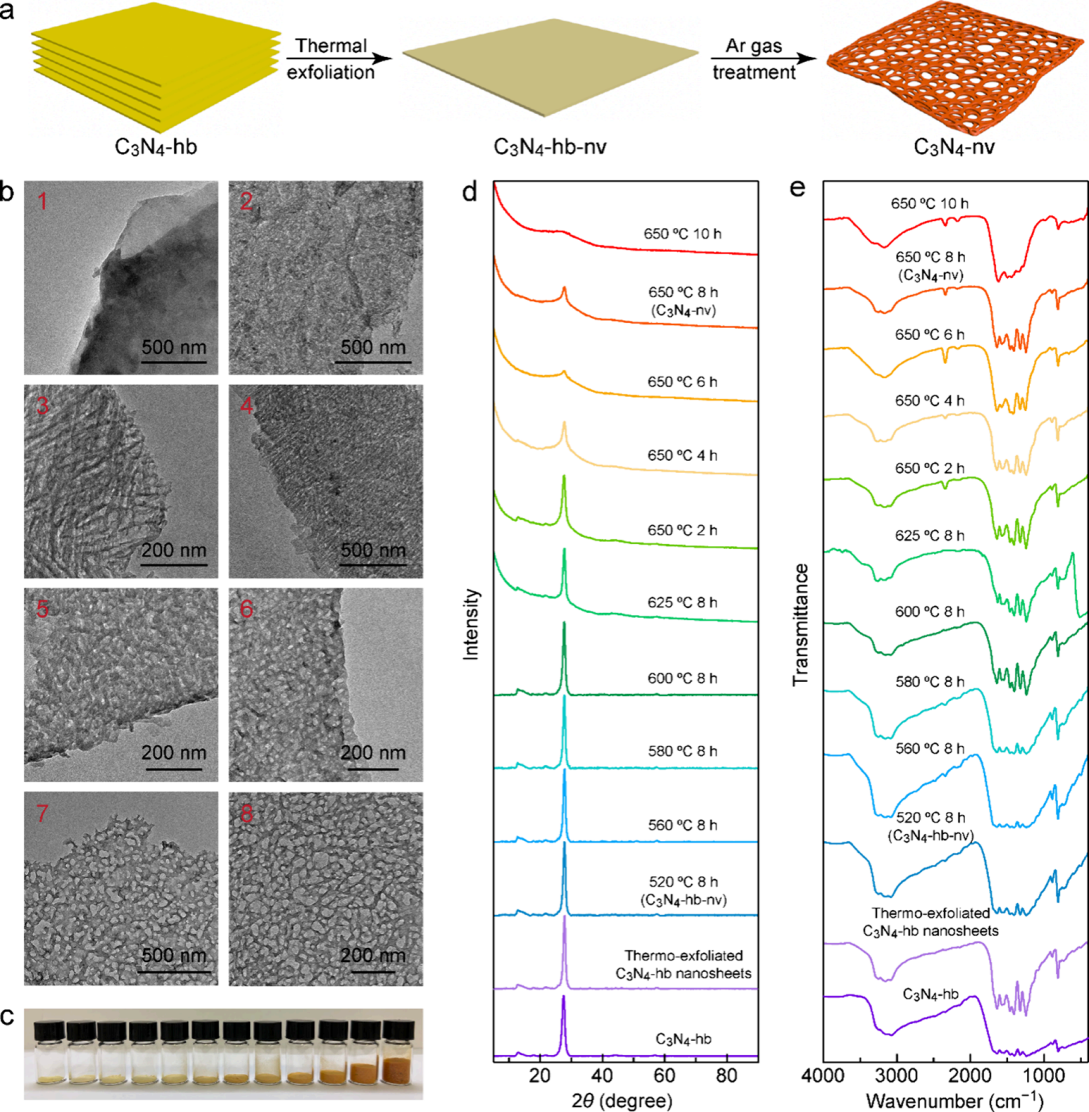
Synthesis and characterization of the photocatalysts. (a) Schematic illustrating the synthetic process of the carbon nitride samples, including C_3_N_4_-hb, C_3_N_4_-hb-nv, and C_3_N_4_-nv. (b) TEM images of the as-prepared carbon nitride samples. 1: C_3_N_4_-hb. 2: thermally exfoliated C_3_N_4_-hb nanosheets. 3: carbon nitride treated at 520 °C for 8 h (C_3_N_4_-hb-nv). 4: carbon nitride treated at 560 °C for 8 h. 5: carbon nitride treated at 600 °C for 8 h. 6: carbon nitride treated at 650 °C for 2 h. 7: carbon nitride treated at 650 °C for 8 h (C_3_N_4_-nv). 8: carbon nitride treated at 650 °C for 10 h. (c) Photograph of the different samples with the same mass. (d) XRD patterns. (e) FTIR spectra.

The intralayer hydrogen bond breaking in carbon nitride was realized by subjecting the material to post-thermal treatment under an inert atmosphere, as illustrated in Figure 2a. The bulk g-C_3_N_4_ (referred as C_3_N_4_-hb) was synthesized through the calcination of melamine in an air environment. The as-obtained C_3_N_4_-hb sample was subsequently ground into powder and further thermally exfoliated to form C_3_N_4_-hb nanosheets. These C_3_N_4_-hb nanosheets were finally subjected to thermal treatment under inert Ar gas at various temperatures and durations. The treatment temperatures ranged from 520 to 650 °C, and the durations ranged from 2 to 10 h. The morphologies of the treated carbon nitride samples exhibit clear changes associated with the disruption of intralayer hydrogen bonds, as revealed by the transmission electron microscopy (TEM) images (Figure 2b and Figure S1). The pristine C_3_N_4_-hb possesses a smooth surface, whereas the thermal treatment induces the formation of abundant pores with relatively uniform sizes. Notably, at 520 °C, the thermal treatment results in the formation of slit holes with uniform widths. These slit holes share one orientation and are nearly parallel to each other. The formation of these slit holes along the strands is attributed to the volume shrinkage of the carbon nitride owing to the partial breaking of the hydrogen bonds between the melon strands. The single orientation of the strands determines the single orientation of the formed slit holes. With increases in the treatment temperature, a higher portion of the intralayer hydrogen bonds are broken. In addition, the interconnected pore walls gradually become thinner as the treatment temperature is increased. However, at temperatures exceeding 650 °C, some pore walls collapse owing to the thermal decomposition of the melon strands. The pore size exhibits slight changes, while the specific surface area gradually increases from 10.4 to 128.6 m^2^ g^–1^ (Figures S2 and S3). This structural modification of C_3_N_4_ results in a significant increase in the sample volume for a given mass (Figure 2c). Moreover, the color of C_3_N_4_ changes from light yellow to dark orange, accompanying the structural modification (Figure 2c), which is advantageous for efficient solar light harvesting.

The evolution of the XRD patterns of the carbon nitride samples with the treatment temperature and duration is displayed in Figure 2d. As mentioned above, the peaks observed at 13.1 and 27.2° can be indexed to the in-plane-ordered tri-*s*-triazine motifs and the interlayer stacking of the aromatic systems, respectively. The peak centered at 13.1° gradually diminishes as the treatment temperature is increased, indicating the disruption of the intralayer periodicity of the tri-*s*-triazine units and the progressive breaking of the intralayer hydrogen bonds. Simultaneously, the peak centered at 27.2° becomes broader and weaker owing to the fluctuation of the intralayer structure and disruption of the periodic stacking of the layers, resulting from the breaking of the intralayer hydrogen bonds. At higher treatment temperatures, the long-range periodicity of C_3_N_4_ is completely disrupted, giving rise to a relatively broad peak in the XRD patterns. To further investigate the chemical structures of the different C_3_N_4_ samples, Fourier transform infrared spectroscopy (FTIR) was employed. All the samples exhibit several similar broad characteristic absorption bands (Figure 2e). The band observed at 808 cm^–1^ is attributed to the breathing mode of the triazine units, while the band ranging from 1200 to 1900 cm^–1^ arises from the stretching vibrations of the aromatic CN heterocycles. The broad absorption band in the range of 2400 to 3650 cm^–1^ is ascribed to the overlapping vibrations of −OH stretching (C–OH groups and adsorbed H_2_O) and the stretching vibrations of the −NH and −NH_2_ groups. The primary change observed after thermal treatment is the emergence of a new peak at 2180 cm^–1^, resulting from the formation of −C≡N groups through the deprotonation of −C–NH_2_.

**Figure 3:**
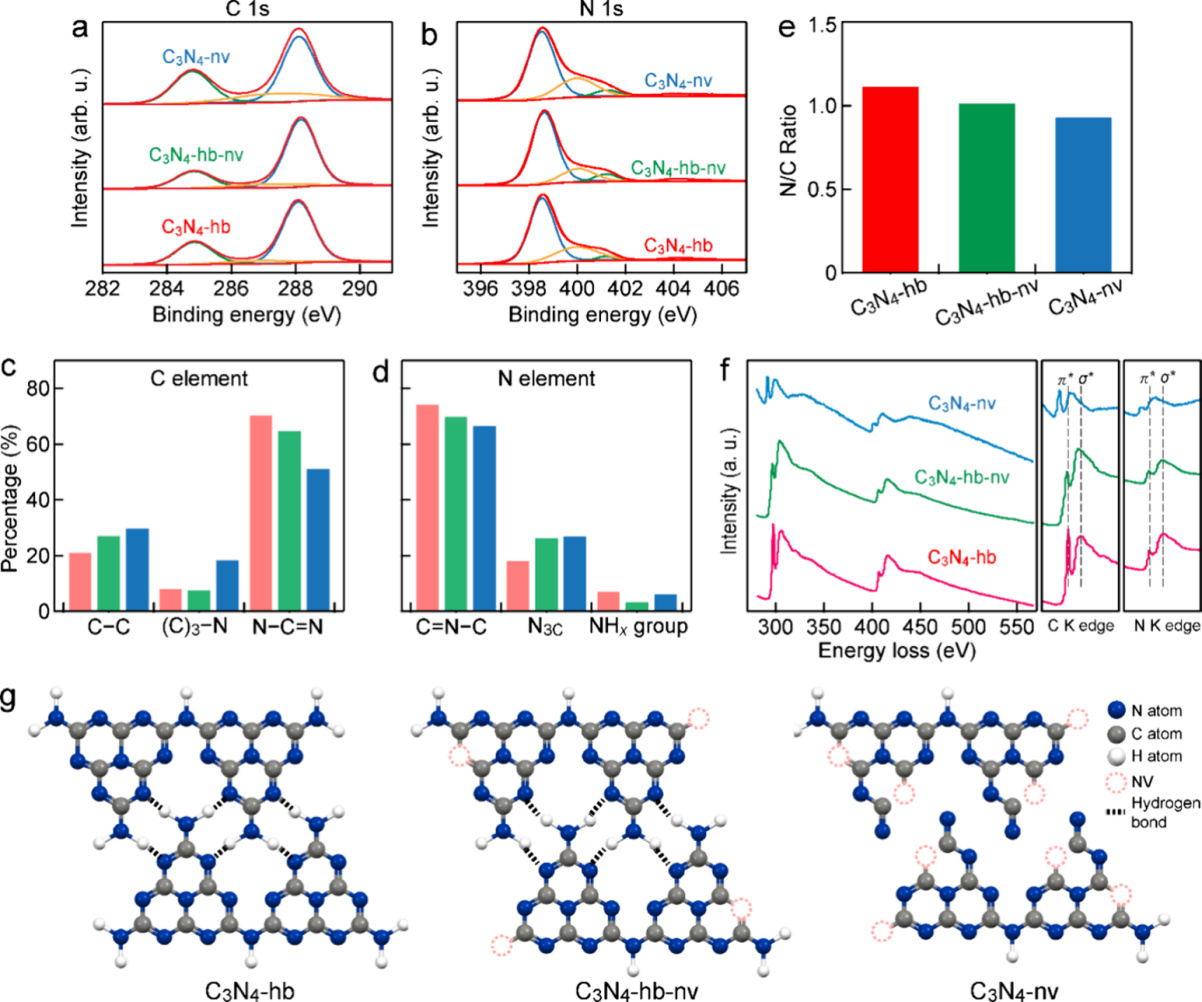
Chemical structures of C_3_N_4_-hb, C_3_N_4_-hb-nv, and C_3_N_4_-nv. (a, b) High-resolution C 1s (a) and N 1s (b) XPS spectra. (c, d) Percentages and ratios of the three peaks in the C 1s (c) and N 1s (d) XPS spectra. (e) Atomic ratios of N:C determined from elemental analysis. (f) EELS results revealing the C K and N K edges with the σ* and π* resonances. (g) Derived chemical structures of C_3_N_4_-hb, C_3_N_4_-hb-nv, and C_3_N_4_-nv.

Based on the distinct characteristics revealed by these samples, three representative samples were selected for further comparison: bulk g-C_3_N_4_ with intralayer hydrogen bonds but no NVs, C_3_N_4_ treated at 520 °C for 8 h with intralayer hydrogen bonds and NVs, and C_3_N_4_ treated at 650 °C for 8 h with NVs but complete hydrogen bond breaking. These samples are referred to as C_3_N_4_-hb, C_3_N_4_-hb-nv, and C_3_N_4_-nv, respectively. Their names were also defined according to the following characterization results. X-ray photoelectron spectroscopy (XPS) was employed to examine their surface compositions. Their XPS spectra all exhibit three distinct sharp peaks centered at ∼289, 400, and 534 eV, which can be ascribed to C 1s, N 1s, and O 1s, respectively (Figure S4). Peaks for NVs were not observed in the XPS spectra, which might be caused by the poor surface charging effect of carbon nitride or the measurement limitation.^38,39^ The C 1s and N 1s peaks were subsequently deconvoluted (Figure 3a,b). The N 1s peaks were deconvoluted into three peaks, which correspond to bicoordinated N atoms (N_2C_), tertiary nitrogen N–(C)_3_ groups (N_3C_), and NH_*x*_ groups. Similarly, the C 1s peaks were also deconvoluted into three peaks, representing C–C, (C)_3_–N, and bicoordinated C atoms (C_2N_). The percentages and ratios of the three peaks in the N 1s and C 1s XPS spectra were determined (Tables S1 and S2). Figure 3c,d shows the relative distributions of the different groups. The percentage of C=N–C in the N 1s peak gradually decreases from C_3_N_4_-hb to C_3_N_4_-hb-nv to further C_3_N_4_-nv, suggesting the progressive destruction of the C=N–C groups upon the thermal treatment. The percentage of the NH_*x*_ groups in the N 1s peak initially decreases in C_3_N_4_-hb-nv and then slightly increases in C_3_N_4_-nv. Nevertheless, the amounts of the NH_*x*_ groups in both C_3_N_4_-hb-nv and C_3_N_4_-nv are lower than that in C_3_N_4_-hb, which indicates a reduction in the quantity of the NH_*x*_ groups as well as their breakdown. The atomic ratio of N:C, as determined by elemental analysis, decreases from 3.36:3 for C_3_N_4_-hb to 3.05:3 for C_3_N_4_-hb-nv and further to 2.80:3 for C_3_N_4_-nv (Figure 3e). It should be noted that the residual NH_*x*_ groups are responsible for the formation of intralayer hydrogen bonds. As the NH_*x*_ groups are more susceptible to destruction compared to N_2C_ and N_3C_, the decreased N:C ratios are attributed to the loss of the NH_*x*_ and C=N–C groups. Therefore, the XRD and XPS results collectively indicate the destruction of the NH_*x*_ and C=N–C groups, accompanied by the breaking of the intralayer hydrogen bonds during thermal treatment. The ^13^C magic-angle spinning nuclear magnetic resonance (MAS NMR) spectra further confirm their chemical structures (Figure S5). Two major peaks located at about 164 and 156 ppm can be observed from the ^13^C MAS NMR spectra. They can be ascribed to the chemical shifts of C_2N-NH*x*_ (C3) and C_3N_ (C2), respectively. These two main peaks are observed for C_3_N_4_-hb, C_3_N_4_-hb-nv, and C_3_N_4_-nv, which confirms that intralayer hydrogen bond breaking does not change the main structure of carbon nitride. In addition, three weak peaks appear at 120 ppm for C1 (−C≡N groups), 169 ppm for C4 (−NH–C≡N), and 172 ppm for C5 (=N–C≡N) on C_3_N_4_-nv. This result indicates that intralayer hydrogen bond breaking leads to the deprotonation of the C–NH_2_ groups and introduce −C≡N groups, which is consistent with the results from FTIR and XPS. Electron energy loss spectroscopy (EELS) measurements were thereafter performed to characterize their chemical structures (Figure 3f). In the EELS spectra of the three samples, both the C K and N K edges show σ* and π* resonances. In the C K edge spectra, two peaks are ascribed to the 1s to σ* and 1s to π* electronic transitions of sp^2^-hybridized carbon. The two peaks in the N K edge spectra is believed to arise from the N atoms sp^2^-hydridized with C atoms and N atoms, respectively.^40^ The resonance peaks of C_3_N_4_-nv are both blueshifted, suggesting that the valence numbers of the sp^2^-hydridized N and C atoms become higher. The increased valence numbers of the sp^2^-hydridized N and C atoms are caused by lower valence electron densities, which indicates electron transfer from the pyridinic nitrogen (sp^2^-hydridized N atoms) to the electron-withdrawing groups, such as −C≡N groups and NVs. Based on these findings, the chemical structures of these three samples can be derived (Figure 3g). In C_3_N_4_-hb and C_3_N_4_-hb-nv, hydrogen bonds are present, while in C_3_N_4_-nv, hydrogen bonds are completely broken. In C_3_N_4_-hb-nv, NVs are formed at the locations of the C=N–C and NH_*x*_ groups, which are not connected by hydrogen bonds. Similarly, in C_3_N_4_-nv, NVs are also formed at the locations of the C=N–C and NH_*x*_ groups, but in this case, C=N–C and NH_*x*_ groups are connected by hydrogen bonds as observed in C_3_N_4_-hb-nv. In addition, −C≡N groups are formed in C_3_N_4_-nv due to the deprotonation of −C–NH_2_.

**Figure 4:**
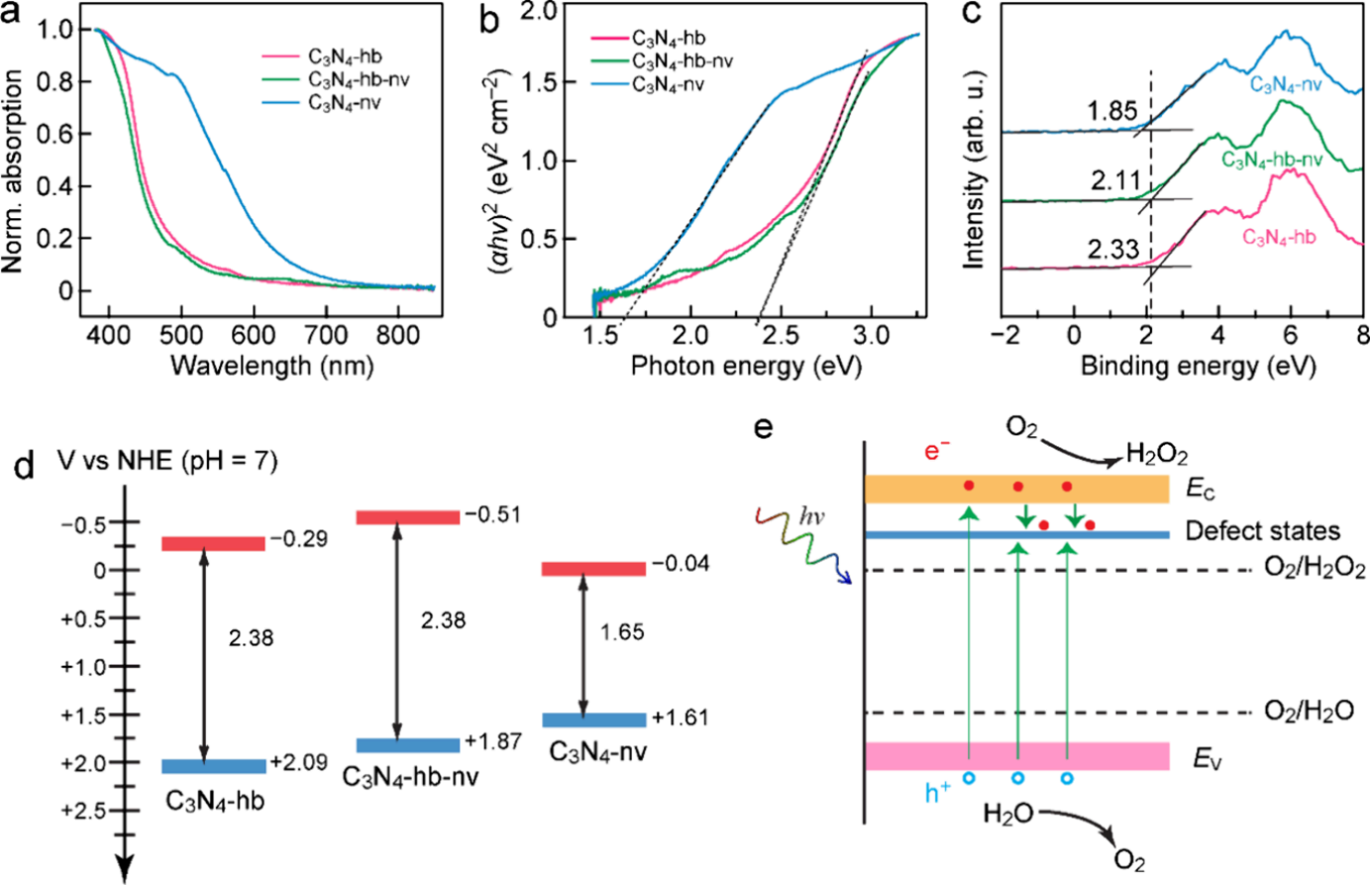
Band structures of C_3_N_4_-hb, C_3_N_4_-hb-nv, and C_3_N_4_-nv. (a) Light absorption spectra. (b) Tauc plots derived from the corresponding light absorption spectra. (c) XPS VB spectra. (d) Determined band-edge positions. (e) Schematic illustrating the electronic band structure of C_3_N_4_-nv in comparison with the redox potentials of the involved reactions.

The chemical structure modification induces significant changes in the electronic structure of the carbon nitride samples, thus resulting in alterations in light absorption, charge transport, and charge separation. The light absorption spectra of the different C_3_N_4_ samples are depicted in Figure 4a and Figure S6. In comparison with C_3_N_4_-hb, the thermally treated samples exhibit substantially reinforced light absorption, with a pronounced redshift of the absorption edge into the near-infrared region as the treatment temperature and duration are increased. Moreover, in C_3_N_4_-hb, the light absorption is attributed to the intrinsic electronic transition from π to π*. The enhanced π–π* electronic transition can be ascribed to the expanded π-conjugated aromatic framework and better packing of the joint heptazine system. Notably, in C_3_N_4_-nv, a new absorption peak around 490 nm emerges, which is attributed to the *n* to π* electronic transition of the lone pair electrons at the defect sites (−C≡N) (Figure 4a).^41^ Their electronic band gap was determined using the Tauc relationship derived from the absorption spectra (Figure 4b). The calculated band gaps are 2.38, 2.38, and 1.65 eV for C_3_N_4_-hb, C_3_N_4_-hb-nv, and C_3_N_4_-nv, respectively. The XPS valence band (VB) spectra in Figure 4c display the well-determined VB edge positions for C_3_N_4_-hb, C_3_N_4_-hb-nv, and C_3_N_4_-nv at 2.33, 2.11, and 1.85 eV, respectively. The VB potential values show a gradually decreasing tendency. The VB edge positions were further converted to potentials relative to the normalized hydrogen electrode (NHE) according to
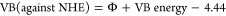
5where Φ is the work function of the XPS analyzer and 4.44 eV represents the work function of the NHE. The determined VB edge potentials against the NHE are +2.09, +1.87, and +1.61 V for C_3_N_4_-hb, C_3_N_4_-hb-nv, and C_3_N_4_-nv, respectively (Figure 4d).

Combined with the band gap, the conduction band (CB) edge positions of the three photocatalysts were determined to be −0.29, −0.51, and −0.04 V against NHE. The obtained CB positions are in good agreement with the values obtained from the Mott–Schottky curves except for C_3_N_4_-nv (Figure S7). The reason why the CB from the Mott–Schottky measurement for C_3_N_4_-nv is different from that obtained from the XPS VB spectra might be the inaccuracy of the Mott–Schottky measurement, which might in turn be caused by the preparation of electrodes and the measurement setup. The XPS VB measurement is highly believed to be more reliable for determining band-edge positions. In addition, the band-edge position obtained from the XPS VB spectra is also in good agreement with the required band-edge position for H_2_O_2_ production from the one-step two-electron ORR. The CB edge is more negative than the redox potential of O_2_/H_2_O_2_, while the VB edge is more positive than the redox potential of O_2_/H_2_O (Figure 4e). This observation indicates that the photogenerated charge carriers in C_3_N_4_-nv possess sufficient energy to drive both the two-electron ORR and four-electron WOR.

**Figure 5:**
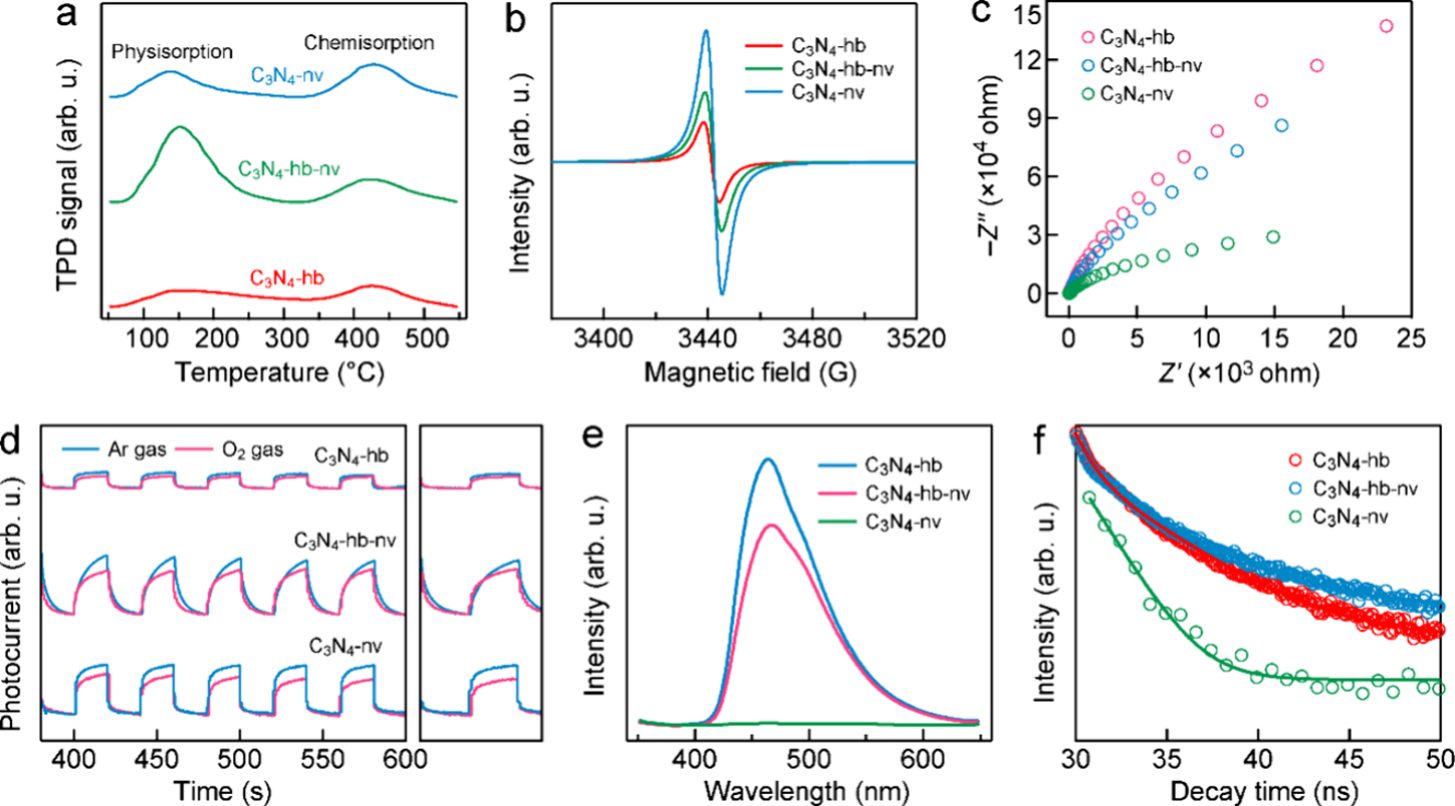
Properties of C_3_N_4_-hb, C_3_N_4_-hb-nv, and C_3_N_4_-nv. (a) O_2_ TPD profiles. (b) EPR spectra. (c) EIS Nyquist plots. (d) Photocurrent responses in Ar and O_2_ atmospheres with the visible-light irradiation switched on and off repeatedly. (e) Steady PL spectra with excitation at 330 nm. (f) Transient PL decay spectra with excitation at their absorption edge. The red, blue, and green solid lines are the fitting results of the decay spectra.

The chemisorption of O_2_ molecules is an essential step in the ORR process. To assess the O_2_ adsorption capability, O_2_ temperature-programmed desorption (O_2_-TPD) was performed (Figure 5a). Before the TPD measurement, thermogravimetric analysis (TGA) was performed to determine the appropriate temperature for the TPD setup (Figure S8). For all the three samples, two desorption peaks were observed. The lower-temperature desorption peak corresponds to physically adsorbed O_2_, while the higher-temperature one belongs to chemically adsorbed O_2_. The chemisorption of O_2_ on C_3_N_4_-hb is originated from the formation of 1,4-endoperoxide species through the adsorption of O_2_ on carbon nitride, as reported in previous studies.^25,29^ In comparison with C_3_N_4_-hb, both the intensities of the physisorption and chemisorption peaks on C_3_N_4_-hb-nv and C_3_N_4_-nv are increased. Among them, C_3_N_4_-hb-nv exhibits the strongest physisorption, which might be associated with its superior pore size and volume. C_3_N_4_-nv possesses the largest relative intensity ratio of chemisorption to physisorption, which is consistent with the presence of rich defects, including both NVs and −C≡N groups, formed in C_3_N_4_-nv. In contrast, C_3_N_4_-hb-nv only has a small number of NVs, which leads to its weaker chemisorption.

In addition, NVs were confirmed to exist in C_3_N_4_-hb-nv and C_3_N_4_-nv by XPS spectra (Figure 3). To further investigate the NVs, low-temperature electron paramagnetic resonance (EPR) analysis was conducted. A characteristic EPR signal with a *g* factor of 2.003 was observed (Figure 5b), indicating the existence of NVs. Notably, C_3_N_4_-nv exhibits the strongest EPR signal while C_3_N_4_-hb displays the weakest signal. The NVs are capable of electron redistribution to the adjacent C atoms within the delocalized π-conjugated network. The increase in the EPR signal with the increase in the treatment temperature indicates an elevated concentration of unpaired electrons within the aromatic rings. This higher concentration of delocalized unpaired electrons facilitates electron transfer to reactive adsorbates in the catalytic reactions. The abundant NVs offer plentiful chemisorption and activation sites for O_2_ molecules, promoting O_2_ reduction by electrons trapped at the NV sites.

The higher concentration of delocalized electrons leads to reduced charge transfer resistances, as revealed by the smaller impedance arc radii observed in the electrochemical impedance spectra (EIS, Figure 5c). The photocurrent responses of the three photocatalyst samples were measured under visible light in both Ar and O_2_ atmospheres (Figure 5d). The absolute photocurrents increase in the order of C_3_N_4_-hb, C_3_N_4_-hb-nv, and C_3_N_4_-nv in both Ar and O_2_ atmospheres, which is consistent with the impedance differences. Compared with those under an Ar atmosphere, the photocurrents of C_3_N_4_-hb, C_3_N_4_-hb-nv, and C_3_N_4_-nv are reduced by 14.1, 15.5, and 54.9% in the O_2_ atmosphere, respectively (Figure 5d, right side). The increased photocurrent differences between Ar and O_2_ are ascribed to the enhanced electron consumption by O_2_ molecules at the active sites. The presence of NVs is beneficial for interfacial electron transfer from the photocatalyst to the activated O_2_ molecules. To investigate charge carrier recombination, photoluminescence (PL) measurements were performed (Figure 5e,f). Compared with C_3_N_4_-hb, C_3_N_4_-hb-nv shows a clear decrease in PL intensity and C_3_N_4_-nv exhibits negligible PL emissions (Figure 5e). This result suggests that radiative recombination of charge carriers is greatly inhibited in C_3_N_4_-hb-nv and C_3_N_4_-nv owing to the presence of the NVs and −C≡N groups. Moreover, transient PL spectra were obtained by exciting at their absorption edge and monitoring at their emission peaks. As shown in Figure 5f and Table S3, the PL lifetime of C_3_N_4_-nv (64.79 ns) is nearly 10 times longer than that of C_3_N_4_-hb (6.38 ns) and C_3_N_4_-hb-nv (13.73 ns). Overall, C_3_N_4_-nv with complete hydrogen bond breaking and abundant NVs shows greatly enhanced charge transfer and separation efficiency behaviors.

### H_2_O_2_ Photosynthesis

**Figure 6:**
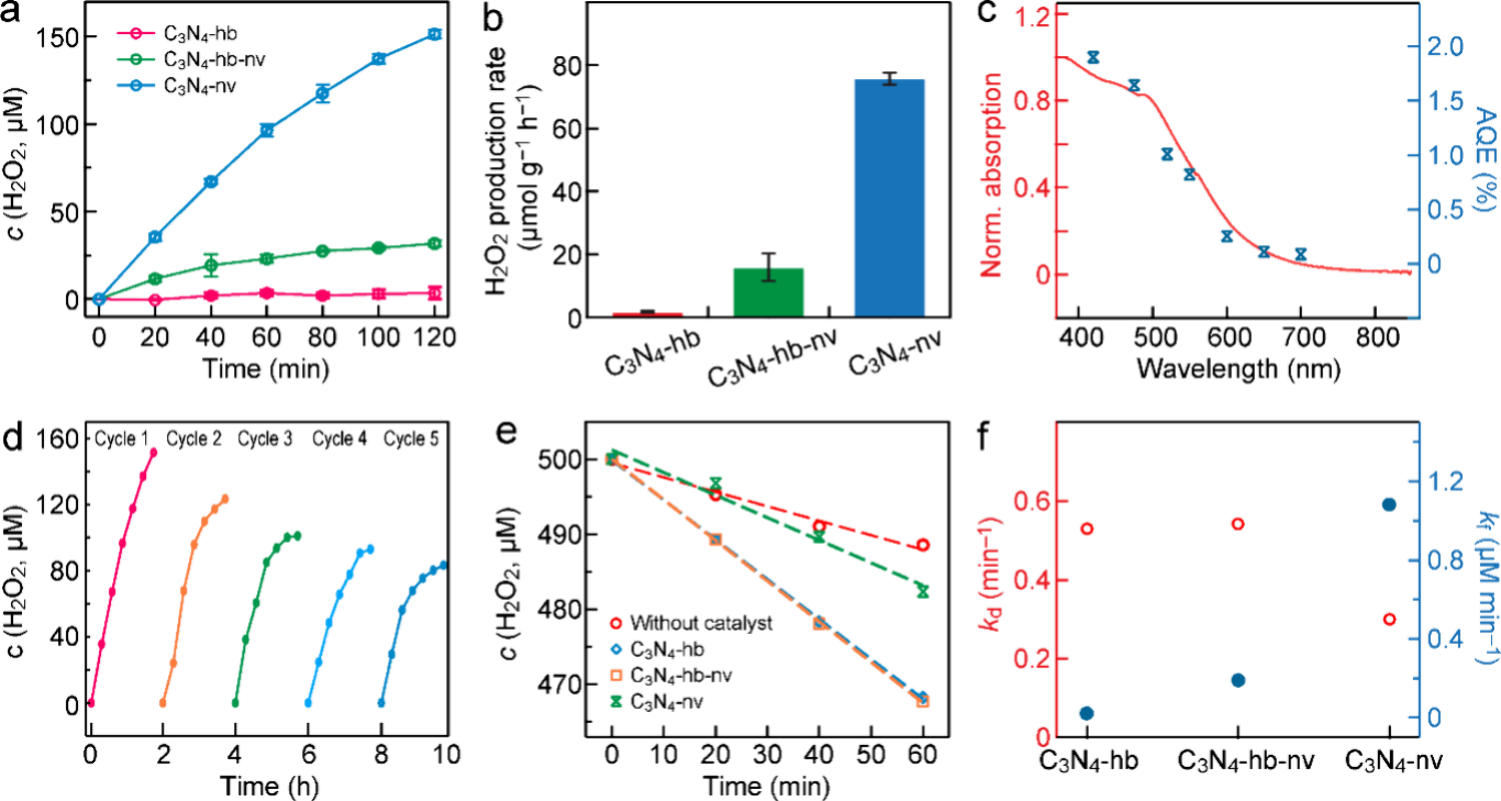
Photocatalytic H_2_O_2_ production of C_3_N_4_-hb, C_3_N_4_-hb-nv, and C_3_N_4_-nv. (a) Time courses of the generated H_2_O_2_ concentrations under visible-light irradiation (λ > 420 nm). (b) H_2_O_2_ production rates. The error bars in (a, b) represent one standard deviation. (c) Light absorption (left axis) and AQE action (right axis) spectra of C_3_N_4_-nv. (d) Cycling tests for H_2_O_2_ production with C_3_N_4_-nv. (e) Photocatalytic decomposition of H_2_O_2_ with C_3_N_4_-hb, C_3_N_4_-hb-nv, and C_3_N_4_-nv under visible-light irradiation. (f) H_2_O_2_ formation rate constant (*k*_f_) and decomposition rate constant (*k*_d_) over C_3_N_4_-hb, C_3_N_4_-hb-nv, and C_3_N_4_-nv.

The successful synthesis of photocatalysts with abundant active sites, increased specific surface areas, intensified charge carrier generation and transfer, enhanced light absorption, and appropriate band gaps enables the exploration of their H_2_O_2_ photosynthetic activities under visible-light irradiation. H_2_O_2_ photosynthesis was evaluated with a home-built photocatalytic reactor (Figure S9), with sufficient gaseous O_2_ supply in aqueous solutions. The produced H_2_O_2_ amount was determined using the well-established cerium sulfate Ce(SO_4_)_2_ chromogenic method, for which the calibration relationship between the peak absorbance and the H_2_O_2_ concentration was predetermined (Figure S10). The pristine C_3_N_4_-hb sample displays a negligible H_2_O_2_ synthesis activity, while the thermally treated carbon nitride samples present significantly increased H_2_O_2_ production rates. The H_2_O_2_ production rate is dependent on the treatment temperature and time (Figure S11). The performance of the three representative photocatalysts is shown in Figure 6a,b. Among all the samples, C_3_N_4_-nv gives the optimal H_2_O_2_ photosynthetic rate of 75.66 μmol g^–1^ h^–1^, which is nearly 45 times higher than that of the pristine C_3_N_4_-hb sample. In contrast, C_3_N_4_-hb-nv exhibits an H_2_O_2_ production rate of 15.90 μmol g^–1^ h^–1^, nearly 9.5 times higher than that of the pristine C_3_N_4_-hb sample. The enhanced photocatalytic activities of C_3_N_4_-hb-nv and C_3_N_4_-nv are associated with their improved light-harvesting capacities, increased specific surface areas, and abundant reactive sites. Control experiments confirm that H_2_O_2_ cannot be generated in the absence of any one of the following conditions: the catalyst, light irradiation, or O_2_ (Figure S12).

The dependence of the H_2_O_2_ photosynthesis activities on the light absorption of the photocatalysts was further studied by acquiring the action spectrum (Figure 6c and Table S4). The H_2_O_2_ photosynthesis was performed with C_3_N_4_-nv under monochromatic light irradiation at different wavelengths. The apparent quantum efficiency (AQE) of the H_2_O_2_ photosynthesis at each wavelength was determined from the ratio of the electron number involved in the H_2_O_2_ photosynthesis to the incident photon number. The electron number involved in the photocatalytic reaction is twice the number of the produced H_2_O_2_ molecules. The variation trend of the AQEs is in good agreement with the absorption spectrum of C_3_N_4_-nv. The AQE reaches as high as 1.90% at 420 nm. The LCCE is also used to evaluate the photon utilization and light energy conversion efficiency. A high LCCE value of 3.85% was obtained with C_3_N_4_-nv for the H_2_O_2_ photosynthesis in pure water under visible-light irradiation. It is the highest in comparison with those reported recently (Table S5). The stability of the C_3_N_4_-nv photocatalyst was evaluated by performing the H_2_O_2_ photosynthesis in five cycles. The H_2_O_2_ synthesis rate after five cycles with each photocatalytic reaction time of 120 min retained ∼55% of that of the original photocatalyst (Figure 6d). The C_3_N_4_-nv photocatalyst after reaction was therefore characterized (Figure S13). The FTIR spectra show that the peak at 2180 cm^–1^, resulting from the formation of −C≡N groups, became weaker after reaction. This means that the −C≡N groups might be destructed after reaction. The EPR spectra also reveal a decrease in the concentration of NVs after reaction. The sample turned from orange to light yellow, suggesting that their light absorption decreased after reaction. All the results for the catalyst characterization after reaction show that the catalyst deactivated after reaction. The active sites might be attacked by hot electrons, hot holes, and reactive oxygen species, such as hydroxyl radicals.^42^ Moreover, the mass loss caused by centrifugation after each cycle can also lead to the decrease in the photocatalytic activity.

The photostationary concentration of H_2_O_2_ is known to be determined by the competition between the formation rate (*k*_f_) and decomposition rate (*k*_d_) of H_2_O_2_ over the catalyst. The overall H_2_O_2_ photosynthesis can be calculated by
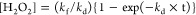
6where the reaction kinetics was acquired by assuming the corresponding zeroth order for *k*_f_ and first-order reaction for *k*_d_ (Figure 6e,f and Table S6). In terms of the fitting results, C_3_N_4_-nv exhibits a larger *k*_f_ and smaller *k*_d_, which is consistent with its higher overall H_2_O_2_ photosynthesis rate than the other two counterparts.

### Understanding the Mechanism of the H_2_O_2_ Photosynthesis

**Figure 7:**
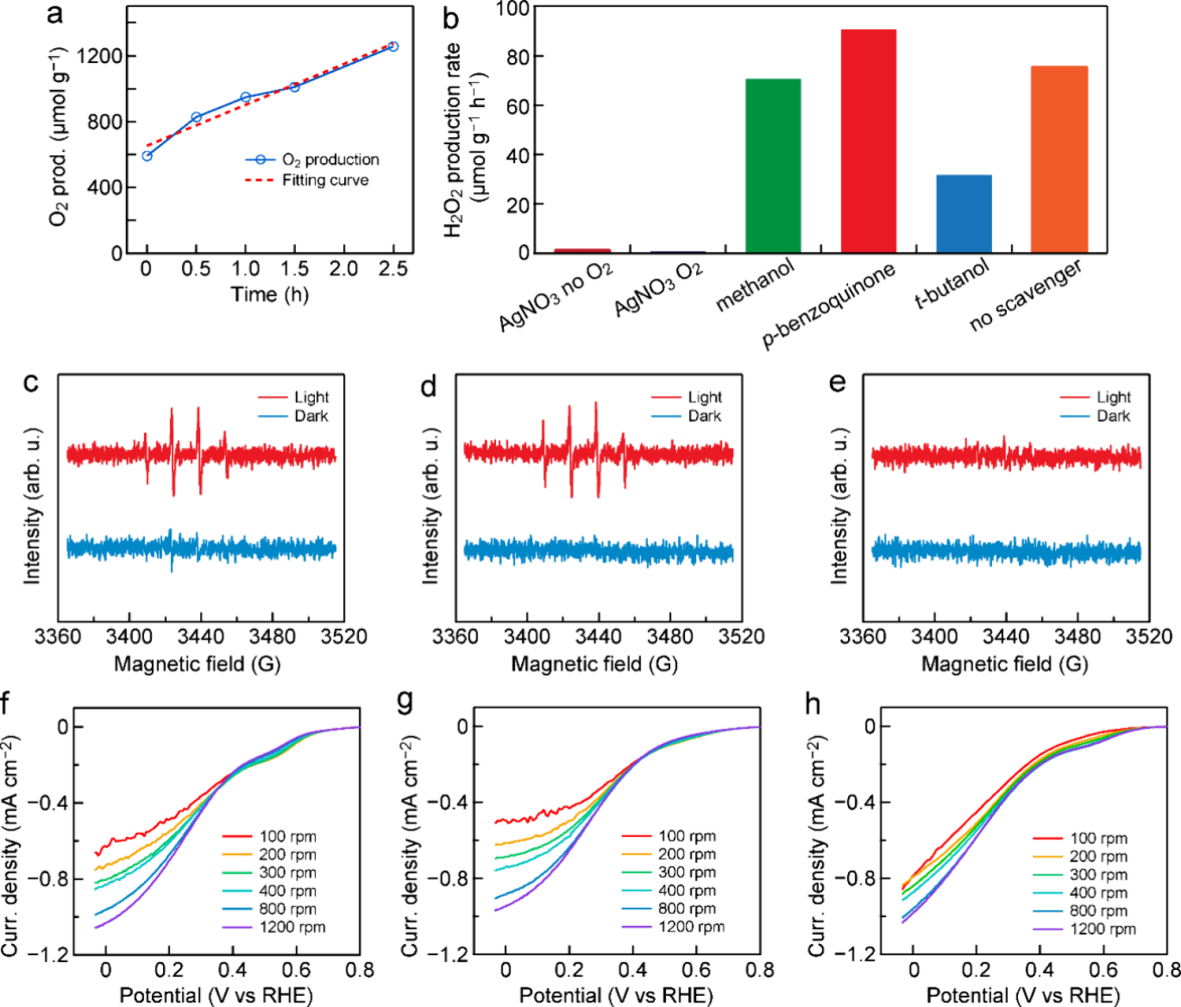
Mechanism investigation. (a) Oxidation product detection by gas chromatography. (b) Photocatalytic H_2_O_2_ production rates in the presence of different scavenger agents under visible-light irradiation. (c–e) EPR spectra of •OH generated with C_3_N_4_-hb, C_3_N_4_-hb-nv, and C_3_N_4_-nv under visible-light irradiation, respectively. (f–h) Linear sweep voltammetry curves of C_3_N_4_-hb, C_3_N_4_-hb-nv, and C_3_N_4_-nv measured on RDE at different rotating speeds, respectively.

In the conducted photocatalytic experiments, H_2_O_2_ was detected as a product only upon the addition of O_2_ gas, indicating that it can be the reduction product from O_2_ by hot electrons. The oxidation product by hot holes, on the other hand, is predicted to be O_2_ gas. To confirm the formation mechanisms of these two redox products in the half-redox reactions, separate experiments were carried out. The oxidation product was confirmed with C_3_N_4_-nv in an aqueous solution in the presence of AgNO_3_ as an electron acceptor and saturated Ar gas bubbling under visible-light irradiation. The gaseous product was detected by gas chromatography with increasing reaction time (Figure S14). The produced O_2_ gas increased in amount with the reaction time, confirming that the oxidation product is O_2_ from H_2_O molecules (Figure 7a). During this process, C_3_N_4_-nv shows a negligible H_2_O_2_ production rate (Figure 7b), indicating that H_2_O_2_ is produced through the reduction of O_2_ by the photoexcited electrons instead of the oxidation of H_2_O molecules. O_2_ gas was therefore produced from the water oxidation by hot holes. Introducing a hole scavenger agent, such as methanol, negligibly affects the H_2_O_2_ production rate on C_3_N_4_-nv, further confirming that H_2_O_2_ is not produced from the water oxidation but from the oxygen reduction. After the introduction of methanol, a little increase in the H_2_O_2_ production rate can sometimes be observed and this might originate from the increased charge carrier transfer. Moreover, possible intermediate radicals involved in the H_2_O_2_ synthesis were probed to determine the reaction path (Figure 7b). Superoxide radicals are considered as potential intermediate species in H_2_O_2_ synthesis, which can be captured by *p*-benzoquinone. However, the introduction of *p*-benzoquinone leads to a little increase in the H_2_O_2_ production rate, indicating that the reduction of O_2_ to H_2_O_2_ undergoes a direct one-step two-electron reduction process without the assistance of superoxide radicals. The increase is highly believed to come from the inhibition of C_3_N_4_-nv degradation by *p*-benzoquinone. *p*-Benzoquinone might protect the active sites of C_3_N_4_-nv from being attacked by reactive oxygen species. Furthermore, hydroxyl radicals also play a crucial role in the H_2_O_2_ production rate. The decomposition of H_2_O_2_ leads to the generation of hydroxyl radicals, as shown in the following equation:
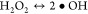
7

If the hydroxyl radicals on the right side of the equation are consumed, the forward reaction will be promoted, leading to a decrease in the gross H_2_O_2_ concentration as well as the net H_2_O_2_ production rate. The consumption of hydroxyl radicals can effectively be achieved by *t*-butanol, a chemical known to trap these radicals. As a result, the introduction of *t*-butanol reduces the production rate of H_2_O_2_ (Figure 7b).

To further confirm whether hydroxyl radicals and superoxide radicals exist or not, EPR spectroscopy was also performed (Figure 7c–e and Figure S15). The results indicate the absence of superoxide radicals on the three representative catalysts, implying that they are not intermediate species in the oxygen reduction to H_2_O_2_. These EPR results align with the experimental results of the superoxide radical trapping with the scavenger agents in Figure 7b. C_3_N_4_-hb, C_3_N_4_-hb-nv, and C_3_N_4_-nv all pass through the one-step two-electron reduction pathway without the generation of the intermediate superoxide radicals. As described in the Introduction section, the formation of 1,4-endoperoxide species on the C_3_N_4_-hb surface suppresses the undesired one-electron ORR to •OOH but preferentially accelerates the selective two-electron ORR to H_2_O_2_. The selectivity of the two-electron ORR toward H_2_O_2_ production over C_3_N_4_-hb is therefore high.^28^ The chemical structure of C_3_N_4_-hb-nv shows that it also possesses the tri-*s*-triazine units connected through planar amino groups (Figure 3g). Therefore, 1,4-endoperoxide species should also form on C_3_N_4_-hb-nv, which can suppress the undesired one-electron ORR to •OOH but preferentially accelerate the selective two-electron ORR to H_2_O_2_. C_3_N_4_-hb-nv should also show a high selectivity of the two-electron ORR toward H_2_O_2_ production, although C_3_N_4_-hb-nv has the suitable band position for the generation of superoxide radicals. In contrast, hydroxyl signals are significantly decreased to a nearly negligible level in C_3_N_4_-nv compared with the other two samples (Figure 7c–e). This observation suggests that the H_2_O_2_ decomposition is greatly inhibited for C_3_N_4_-nv, which agrees well with the lower *k*_d_ values shown in Figure 6f.

Overall, O_2_ gas is reduced into H_2_O_2_ through a one-step two-electron reduction reaction. The average electron transfer number (*n*) involved in the ORR process in the three samples was studied in the O_2_-saturated electrolyte by use of a rotating disk electrode (RDE) at different rotation speeds (Figure 7f–h). The diffusion-limited current density rises with the increase in the rotation speed due to the enhanced oxygen reduction kinetics with faster O_2_ diffusion. The electron transfer number can be calculated from the Koutecký–Levich plots derived from the RDE measurements at different rotation speeds (Figure S16). The electron transfer numbers were determined to be 2.272, 1.804, and 2.001 at 0.25 V vs RHE for C_3_N_4_-hb, C_3_N_4_-hb-nv, and C_3_N_4_-nv, respectively. The H_2_O_2_ formation catalyzed by C_3_N_4_-hb, C_3_N_4_-hb-nv, and C_3_N_4_-nv is therefore dominated by the one-step two-electron ORR pathway.

**Figure 8:**
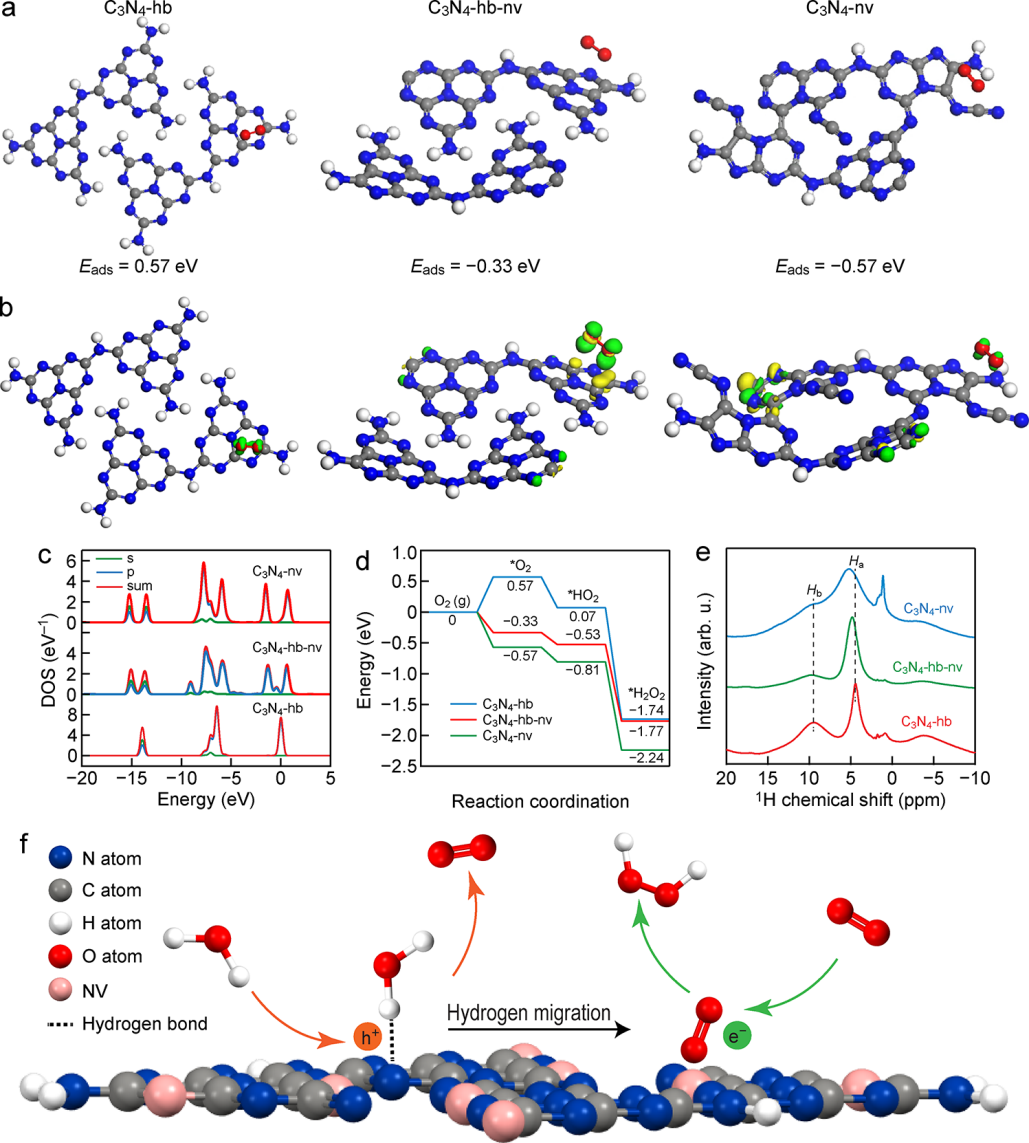
Mechanism investigation by DFT calculations. (a) Adsorption configurations of an O_2_ molecule on the surface. The adsorption energy is shown at the bottom of the adsorption configuration. (b) CDD of adsorbed O_2_. (c) LDOS for the O_2_ molecule after adsorption on the surface of C_3_N_4_-hb, C_3_N_4_-hb-nv, and C_3_N_4_-nv. (d) Energy diagram for the H_2_O_2_ synthesis on the different photocatalysts. (e) Solid-state ^1^H MAS NMR spectra. (f) Proposed reaction mechanism. The blue, gray, white, red, and pink spheres represent the N, C, H, O atoms, and NVs, respectively.

To obtain deeper insights into the reaction mechanism, DFT calculations were further performed. The adsorption and activation of O_2_ on the catalyst surface is a key step in two-electron ORR. The adsorption configurations of an O_2_ molecule on the C_3_N_4_-hb, C_3_N_4_-hb-nv, and C_3_N_4_-nv models are shown in Figure 8a. The O_2_ molecule was found to adsorb on the tri-*s*-triazine unit of C_3_N_4_-hb, on the NV of C_3_N_4_-hb-nv, and on the NV and −C≡N group of C_3_N_4_-nv. The adsorption of O_2_ on C_3_N_4_-hb is determined to be an endothermic process, indicating that the adsorption of O_2_ on C_3_N_4_-hb is unstable. In contrast, C_3_N_4_-hb-nv and C_3_N_4_-nv produce stable adsorption of O_2_, with adsorption energies of −0.33 and −0.57 eV, respectively. Hence, the existence of NVs and −C≡N groups promotes the adsorption of O_2_, which facilitates the subsequent reduction of O_2_. The charge density difference (CDD) and localized density of states (LDOS) were employed to study the electronic structure changes after O_2_ adsorption. The CDD analysis shows that the adsorption of O_2_ on C_3_N_4_-hb-nv and C_3_N_4_-nv results in more prominent charge transfer than on C_3_N_4_-hb (Figure 8b), which is consistent with the variation trend of the adsorption energy. The LDOS analysis demonstrates a substantial splitting of the σ_2p_, π_2p_, and π_2p_* molecular orbitals of O_2_ upon adsorption on C_3_N_4_-hb-nv and C_3_N_4_-nv, while the variation is negligible for O_2_ adsorbed on C_3_N_4_-hb (Figure 8c). This splitting gives rise to purely empty states for the adsorbed O_2_, favoring the acceptance of photoexcited electrons for reduction.

The energy diagrams for the reduction of O_2_ into H_2_O_2_ on C_3_N_4_-hb, C_3_N_4_-hb-nv, and C_3_N_4_-nv are shown in Figure 8d. Owing to the strong adsorption and activation of O_2_, the reduction of O_2_ into H_2_O_2_ is a pure exothermic process on C_3_N_4_-hb-nv and C_3_N_4_-nv. In contrast, C_3_N_4_-hb accounts for an endothermic adsorption step. Hence, C_3_N_4_-hb-nv and C_3_N_4_-nv give rise to higher H_2_O_2_ production rates than C_3_N_4_-hb.

The subsequent hydrogenation of the adsorbed O_2_ molecules, which is also crucial for H_2_O_2_ formation, was further studied through ^1^H nuclear magnetic resonance (^1^H NMR) spectra (Figure 8e). The ^1^H NMR spectra exhibit two prominent peaks at ∼9.1 ppm (H_b_) and 4.0 ppm (H_a_) for the three samples, which are ascribed to amino groups and residual water, respectively. The relative intensity of H_a_:H_b_ increases clearly from C_3_N_4_-hb to C_3_N_4_-hb-nv and further to C_3_N_4_-nv. This indicates the increase of amino groups from C_3_N_4_-hb to C_3_N_4_-hb-nv and further to C_3_N_4_-nv, resulting in the increase of hydrophilicity from C_3_N_4_-hb to C_3_N_4_-hb-nv and further to C_3_N_4_-nv because of the formation of hydrogen bonds between amino groups and H_2_O in the solution. The H_b_ peak is shifted to the lower field, which can be ascribed to the formation of the electron-withdrawing groups of −C≡N. Contact angle measurements also show that C_3_N_4_-nv is superhydrophilic. When a water droplet was released on the surface of C_3_N_4_-nv, it quickly infiltrated the surface and spread out completely, reaching a contact angle of 0° within 0.24 s. In contrast, when a droplet was released on the surface of C_3_N_4_-hb-nv and C_3_N_4_-hb, the contact angle decreased to 36.2 and 48.0° within 0.27 s, respectively. The water droplet spread out completely within a longer time for C_3_N_4_-hb-nv and C_3_N_4_-hb (Figures S17 and S18). The contact angle measurements confirm the increase of hydrophilicity from C_3_N_4_-hb to C_3_N_4_-hb-nv and further to C_3_N_4_-nv. Moreover, the zeta potentials of the C_3_N_4_-hb, C_3_N_4_-hb-nv, and C_3_N_4_-nv samples dispersed in water were further measured (Figure S19). The results show that the hydrogen bond breaking on carbon nitride leads to the formation of more amino groups, which will absorb negatively charged ions in water to form a Stern layer and lead to more negative zeta potentials. Namely, the interaction between carbon nitride and H_2_O molecules becomes stronger with hydrogen bonding breaking on carbon nitride.

According to the combined experimental and theoretical calculation results above, a creditable mechanism centered around C_3_N_4_-nv is put forward (Figure 8f). C_3_N_4_-nv first absorbs photons to generate electron–hole pairs under visible-light irradiation. The photogenerated electrons transfer and accumulate on the defect sites such as the NVs and −C≡N groups. These defect sites are the active sites for the efficient adsorption and activation of O_2_ toward H_2_O_2_. The interaction between the hydrogen-bond-broken carbon nitride surface and O_2_ becomes stronger. The adsorbed and activated O_2_ molecule undergoes a one-step two-electron reduction reaction by the photogenerated electrons to produce H_2_O_2_, while H_2_O molecules are oxidized to O_2_ gas by the photogenerated holes. The H_2_O_2_ decomposition is also inhibited on C_3_N_4_-nv to some extent. Moreover, the hydrogen-bond-broken carbon nitride was found to have more exposed N atoms, which can form hydrogen bonds with H_2_O molecules in the solution. The exposed N atoms serve as proton buffering sites, which facilitates the proton donation. In short, the hydrogen bonds between the exposed N atoms and H_2_O molecules contribute to the enhanced proton-coupled electron transfer. Strong hydrogen bonds increase the proton tunneling dynamics by a factor of 10–10^3^.^36^ Hydrogen migration therefore proceeds from the exposed N atoms to the NVs or −C≡N groups.

## Conclusions

In summary, the interactions between the catalyst surface and reactants, including O_2_ and H_2_O molecules, during H_2_O_2_ photosynthesis are facilitated through the intralayer hydrogen bond breaking in carbon nitride. The intralayer hydrogen bond breaking in carbon nitride is achieved by post-thermal treatment in an inert atmosphere. The as-obtained hydrogen-bond-broken carbon nitride exhibits enhanced light absorption, increased specific surface areas, abundant active sites, and increased charge carrier densities. The hydrogen-bond-broken carbon nitride delivers greatly enhanced photocatalytic H_2_O_2_ production rates and increased AQE and LCCE values. The reaction mechanism study shows that the strengthened interactions between the catalyst surface and reactants play a crucial role. First, the formation of active sites on hydrogen-bond-broken carbon nitride including NVs and cyano groups contributes to the increased adsorption and reduction of O_2_ molecules. O_2_ molecules are reduced by photogenerated electrons in a one-step two-electron reduction pathway. In addition, the interaction between the catalyst surface and H_2_O molecules is also promoted owing to the hydrogen bond formation between the exposed N atoms on hydrogen-bond-broken carbon nitride and H_2_O molecules. The exposed N atoms serve as proton buffering sites, which contribute to the enhancement of proton-coupled electron transfer. Moreover, H_2_O is oxidized to give O_2_ by the photogenerated holes. The decomposition of H_2_O_2_ is also suppressed. Our study provides an attractive method to enhance the interaction between the catalyst surface and reactants and proton donation through the hydrogen bond formation between catalysts and H_2_O molecules.

## Methods

### Catalyst Preparation

The synthesis of the hydrogen-bond-broken carbon nitride samples involved three steps. The first step was the synthesis of bulk g-C_3_N_4_, which was carried out through the thermal condensation of melamine in air in a muffle furnace. Melamine powder was first placed in a lidded alumina crucible. The furnace was heated to 550 °C at a heating rate of 10 °C min^–1^ and then kept at 550 °C for 6 h. Bulk g-C_3_N_4_ was obtained after the muffle furnace cooled down to room temperature.^27^ The second step was the thermal exfoliation of the as-prepared bulk g-C_3_N_4_ into g-C_3_N_4_ nanosheets. The thermal exfoliation process proceeded in air in a muffle furnace, with the bulk g-C_3_N_4_ sample placed in an unlidded alumina crucible and kept at 500 °C for 6 h for complete exfoliation.^43^ The g-C_3_N_4_ nanosheets were obtained after thermal exfoliation. The third step was the thermal treatment of the thermally exfoliated g-C_3_N_4_ nanosheets in Ar gas in a tube furnace. The flow rate of Ar gas was set at 50 mL min^–1^. The treatment temperature ranged from 520 to 650 °C, with the treatment time varied from 2 to 10 h. When the treatment temperature was increased above 650 °C and the treatment time was prolonged, the hydrogen bonds in the thermally exfoliated g-C_3_N_4_ nanosheets were completely destroyed.^34^

### H_2_O_2_ Photosynthesis

The photocatalytic H_2_O_2_ production was performed at room temperature and pressure. A homemade reactor was used. Two openings on the reactor were used for the input and output of O_2_, respectively. For the photocatalytic H_2_O_2_ production, the photocatalyst (50 mg) was dispersed into deionized water (50 mL) in the photocatalytic reactor through mild ultrasonication for 15 min. O_2_ gas was subsequently bubbled into the photocatalyst suspension at a constant flow rate of 50 mL min^–1^ under magnetic stirring. The bubbling process lasted for ∼30 min to ensure the complete adsorption of O_2_ on the photocatalyst and dissolution of O_2_ into the suspension. A 300 W xenon lamp was then turned on to irradiate the reaction solution. Different filters were inserted in the light filter slot to alter the spectral range of the irradiating light. A 420 nm cutoff filter was employed to obtain the visible light, while an AM 1.5G solar light filter was used to simulate sunlight. Various band-pass filters were utilized to create monochromatic light. The light intensity, which was measured with a pyranometer (KIPP&ZONEN CMP3), was controlled by adjusting the intensity button on the xenon light source and the distance between the light source and the reaction solution. The production of H_2_O_2_ with reaction time was monitored by collecting a portion of the reaction solution (3.0 mL) every 20 min, including one portion before the reaction and many portions during the reaction. The collected reaction solution was then immediately centrifuged at 8000 rpm for 2 min to remove the photocatalyst, followed by the filtration with a sterile syringe filter with a 0.22 μm disposable membrane placed inside.

### Evaluation of the Photocatalytic H_2_O_2_ Production

The produced H_2_O_2_ was measured by a traditional cerium sulfate Ce(SO_4_)_2_ colorimetric method.^44^ Ce(SO_4_)_2_ was utilized as an indicator for H_2_O_2_ owing to the reduction of yellow-colored Ce^4+^ ions to colorless Ce^3+^ ions according to the following equation:
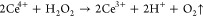
8The concentration of Ce^4+^ can therefore be measured by ultraviolet/visible absorption spectroscopy. The wavelength at 316 nm was the characteristic absorption peak of Ce^4+^ ions in the absorption measurements. The concentration (*c*) of H_2_O_2_ was determined according to the following equation:
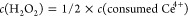
9Specifically, a Ce(SO_4_)_2_ aqueous solution (1 mM) of a yellow transparent color was prepared by dissolving 33.2 mg of Ce(SO_4_)_2_ into a sulfuric acid solution (0.5 M, 100 mL). The calibration curve was obtained by measuring the absorption spectra of Ce(SO_4_)_2_ solutions at different concentrations, which were preprepared by diluting an as-prepared Ce(SO_4_)_2_ solution. According to the linear relationship between the absorption peak intensity and the Ce^4+^ concentration, the H_2_O_2_ concentration of the measured sample solution can be determined.

The production rate of H_2_O_2_ is an intuitive parameter for evaluating the photocatalytic H_2_O_2_ production performance. However, the production rate varied with different conditions, such as the light intensity and the O_2_ gas feeding rate. The AQE and LCCE were therefore employed to evaluate the effects caused by these parameters and quantify the photocatalytic H_2_O_2_ production performance. The AQE and LCCE were used to describe the photocatalytic H_2_O_2_ production performance from microscopic and macroscopic perspectives, respectively. AQE evaluates the incident photon utilization efficiency and is defined as the following equation:
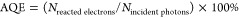
10The reacted electron number (*N*_reacted electrons_) indicates the molar number of the photosynthesized H_2_O_2_, that is, that one mole of H_2_O_2_ represents two moles of transferred electrons in the reaction. Moreover, to obtain the incident light photon number (*N*_incident photons_), monochromic light sources were employed. The incident light photon number was calculated according to the following equation:
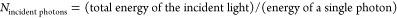
11Besides AQE, LCCE was also employed to quantify the photocatalytic H_2_O_2_ production performance, which is defined according to the following equation:
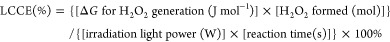
12where Δ*G* is 117 kJ mol^–1^ for the photocatalytic H_2_O_2_ production from water and oxygen gas (O_2_ + 2H_2_O → 2H_2_O_2_). The requirement for the LCCE estimation is that the photocatalytic reaction is free from sacrificial agents. In the LCCE evaluation process, the catalyst (50 mg) and deionized water (50 mL) were added into a home-built reactor, with a cutoff filter (λ > 420 nm) inserted to simulate the visible light. The light intensity was adjusted to 1000 W m^–2^. The cutoff filter (λ > 420 nm) was used to inhibit H_2_O_2_ decomposition.

### Characterization

TEM characterization was performed on FEI Tecnai Spirit operated at 120 kV. XPS characterization was carried out on Thermo Fisher ESCALAB 250Xi. XRD measurements were performed on an X-ray diffractometer (RU-300, Rigaku) with Cu Kα radiation (λ = 1.5406 Å) at room temperature in air. FTIR spectra were recorded on a Thermo Nicolet NEXUS 670 spectrometer. EELS measurements were performed on an FEI Tecnai F20 microscope. Solid-state ^1^H and ^13^C NMR spectra were acquired on Bruker WB 400 M to characterize the molecular structures of the samples by measuring the interaction of nuclear spins under a magnetic field. N_2_ adsorption–desorption isotherms were measured on a Micromeritics ASAP 2020 analyzer. The specific surface areas of the samples were calculated according to the Brunauer–Emmett–Teller (BET) method. Low-temperature EPR spectra were acquired on a Bruker EMX EPR spectrometer (BioSpin GmbH). TGA was conducted on a PerkinElmer system with a heating rate at 10 °C min^–1^. O_2_-TPD was performed on TP-5080 in He gas, with the heating rate set at 10 °C min^–1^. The absorption spectra for the determination of the generated H_2_O_2_ and the absorption spectra of the powder samples were measured on an ultraviolet/visible/near-infrared spectrophotometer (PerkinElmer Lambda 950). Steady-state PL spectra were recorded on a Hitachi F-4600 spectrophotometer. Transient PL spectra were taken on a HORIBA FluoroMax-4 fluorometer. Electrochemical measurements were carried out on an electrochemical workstation (Shanghai Chenhua CHI760E). Contact angle measurements were performed on DataPhysics OCA20. Zeta potentials were measured with DLS-Malvern Instrument Zetasizer ZS90.

### Density Functional Theory Calculations

The DFT calculations were performed using the commercial Vienna Ab initio Simulation Package (VASP).^45,46^ In the calculations, the ion-electron interaction was described by the projector-augmented wave (PAW) method.^47^ The density functional was treated by the generalized gradient density approximation (GGA) with the Perdew–Burke–Ernzerhof (PBE) exchange-correlation potential.^48^ The adsorption configurations of O_2_ on C_3_N_4_-hb, C_3_N_4_-hb-nv, and C_3_N_4_-nv were obtained through complete geometry optimization. The adsorption energy was calculated according to
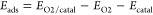
13where *E*_ads_ is the adsorption energy, *E*_O2/catal_ is the energy of the photocatalyst adsorbed with O_2_, *E*_O2_ is the energy of free O_2_, and *E*_catal_ is the energy of the bare photocatalyst.

## Supporting Information Available

TEM images, N_2_ adsorption–desorption isotherms, pore size distributions, XPS spectra, solid-state ^13^C MAS NMR spectra, light absorption spectra, Mott-Schottky curves, and thermogravimetric analysis of the carbon nitride samples; photograph of the reactor; calibration relationship for the determination of the H_2_O_2_ concentrations; photocatalytic H_2_O_2_ production under different conditions; C_3_N_4_-nv photocatalyst before and after reaction; schematic showing the determination of the oxidation product; EPR spectra; Koutecký–Levich plots at different potentials; contact angle images and variations; zeta potentials; binding energies of C 1s and N 1s core electrons; PL decay time values; and determined AQEs and *k*_f_ and *k*_d_ values; comparison of photocatalytic H_2_O_2_ production performances (PDF)
