# Supplementary material for: Promoting Proton Donation through Hydrogen Bond Breaking on Carbon Nitride for Enhanced H2O2 Photosynthesis
Source: ACS Nano. 2024 Jul 26;18(31):20435–48. doi: 10.1021/acsnano.4c04797 (PMC11308773; doi:10.1021/acsnano.4c04797)
Supplement: Supplementary file 1 — nn4c04797_si_001.pdf [file nn4c04797_si_001.pdf]

Supporting Information

Promoting Proton Donation through Hydrogen Bond Breaking on Carbon Nitride for Enhanced H_2O_2 Photosynthesis

Yao Lu,[1,†] Yanzhen Guo,[2,†] Shao Zhang,[1,3] Lejing Li,[4] Ruibin Jiang,[5,] Dieqing Zhang,[3] Jimmy C. Yu,[4] and Jianfang Wang[1,*]*

[1]Department of Physics, The Chinese University of Hong Kong, Shatin, Hong Kong SAR 999077, China

[2]Henan Provincial Key Laboratory of Nanocomposites and Applications, Institute of Nanostructured Functional Materials, Huanghe Science and Technology College, Zhengzhou, Henan 450006, China

[3]The Education Ministry Key Lab of Resource Chemistry, Joint International Research Laboratory of Resource Chemistry, Ministry of Education, and Shanghai Key Laboratory of Rare Earth Functional Materials, College of Chemistry and Materials Science, Shanghai Normal University, Shanghai 200234, China

[4]Department of Chemistry, The Chinese University of Hong Kong, Shatin, Hong Kong SAR 999077, China

[5]School of Materials Science and Engineering, Shaanxi Normal University, Xi'an, Shaanxi 710119, China

[†]Y.L. and Y.Z.G. contributed equally to this work.

Email: rbjiang@snnu.edu.cn; jfwang@phy.cuhk.edu.hk

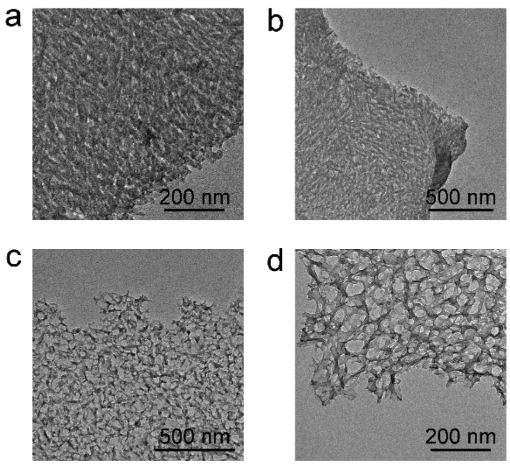

Figure S1. TEM images of the carbon nitride samples. The samples were produced by thermal treatment under different conditions. (a) At 580 °C for 8 h. (b) At 625 °C for 8 h. (c) At 650 °C for 4 h. (d) At 650 °C for 6 h.

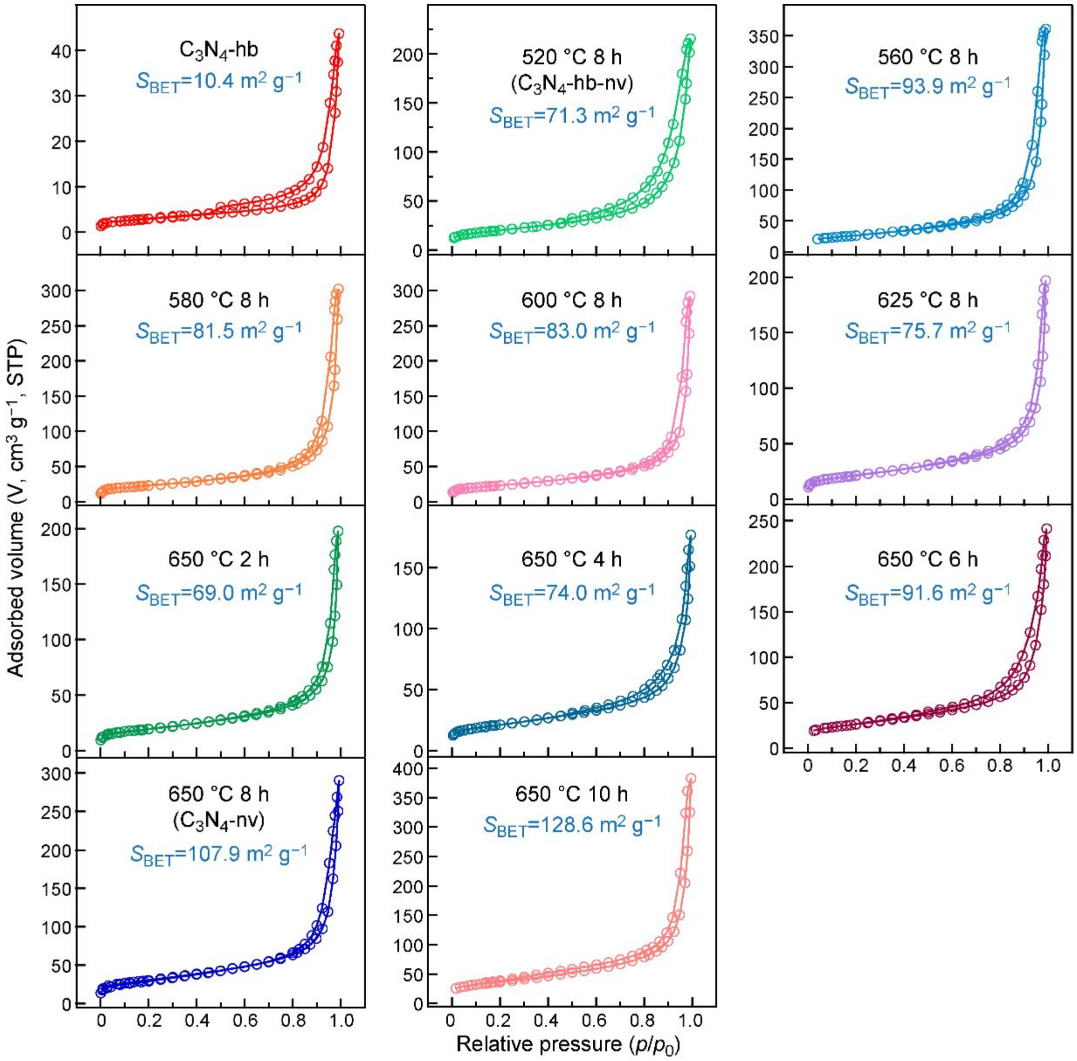

Figure S2. N_2 adsorption-desorption isotherms. All carbon nitride samples were measured.

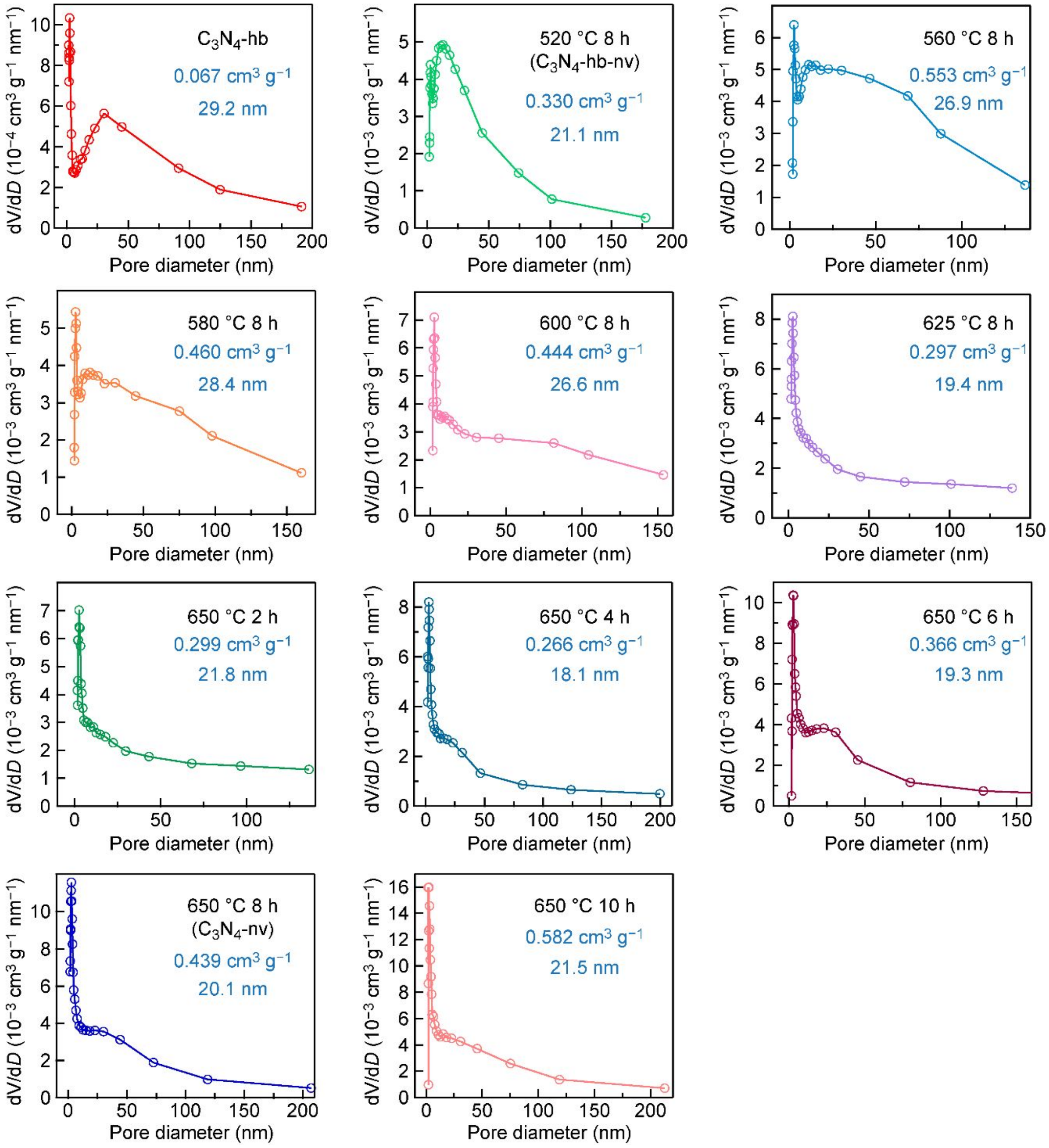

Figure S3. Pore size distributions. All carbon nitride samples were measured.

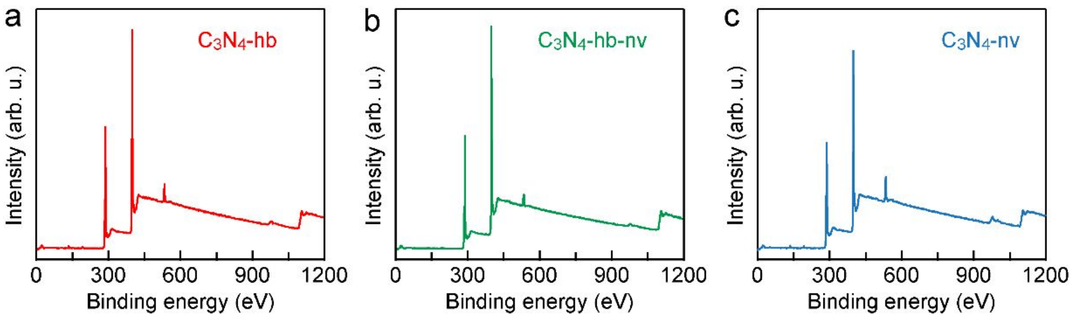

Figure S4. XPS survey spectra. (a) C_3N_4-hb. (b) C_3N_4-hb-nv. (c) C_3N_4-nv.

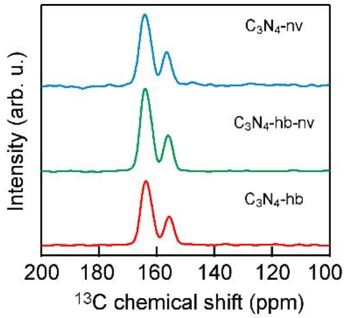

Figure S5. Solid-state ^{13}C MAS NMR spectra. (a) C_3N_4-hb. (b) C_3N_4-hb-nv. (c) C_3N_4-nv.

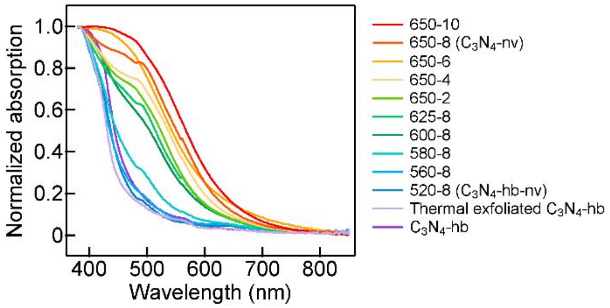

Figure S6. Light absorption spectra of all the samples.

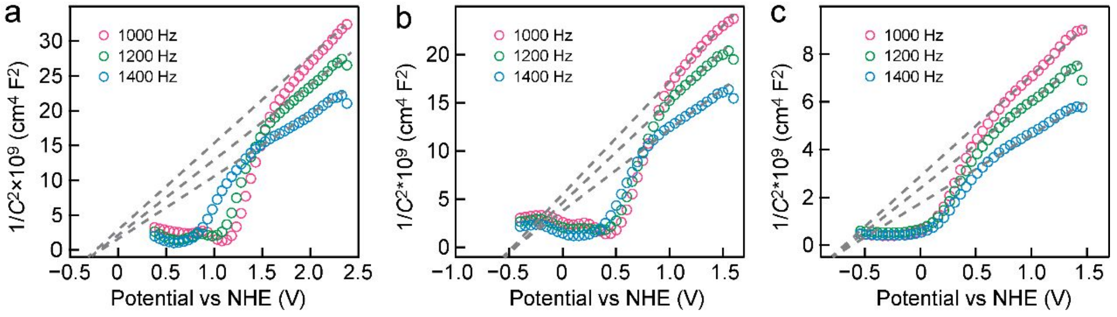

Figure S7. Mott-Schottky curves. (a) C_3N_4-hb. (b) C_3N_4-hb-nv. (c) C_3N_4-nv.

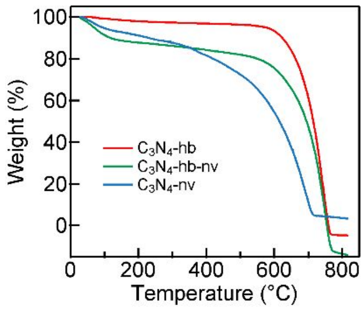

Figure S8. Thermogravimetric analysis of C_3N_4-hb, C_3N_4-hb-nv, and C_3N_4-nv.

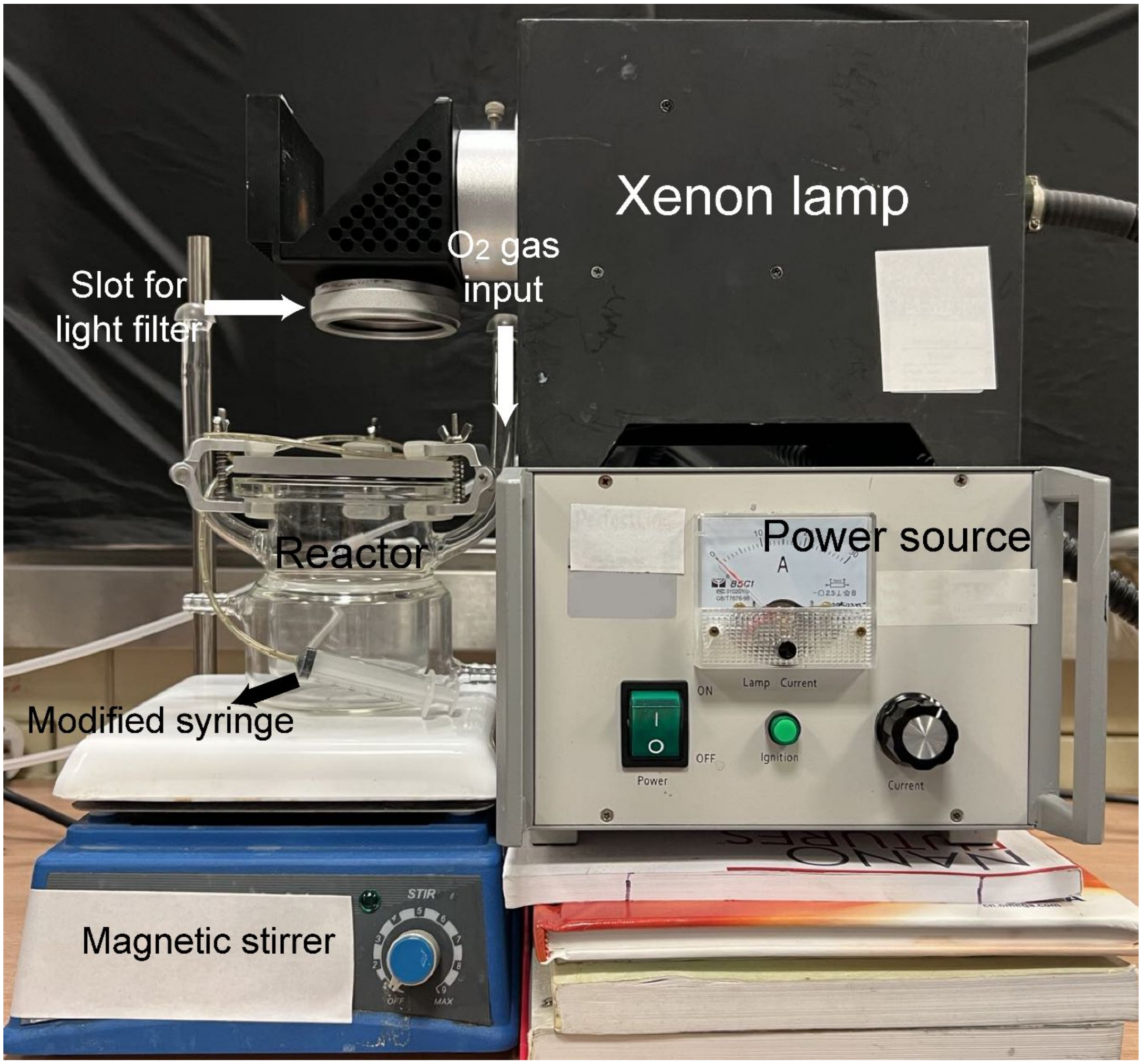

Figure S9. Photograph of the home-built reaction setup.

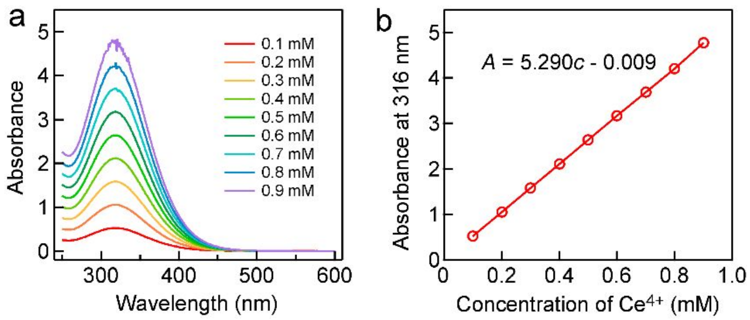

Figure S10. Dependence of the absorbance on the Ce^{4+} concentration. (a) Absorption spectra of $Ce(SO_4)_2$ solutions with different concentrations. (b) Corresponding linear relationship between the absorption peak at 316 nm and the Ce^{4+} concentration.

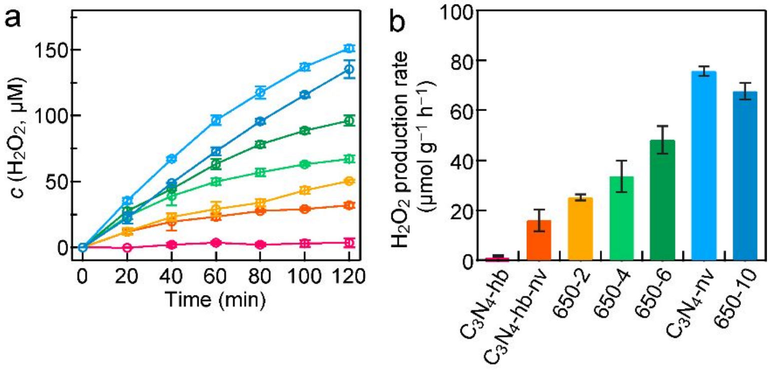

Figure S11. Photocatalytic H_2O_2 production. The photocatalyst samples were tested under visible light irradiation ($\lambda > 420$ nm). (a) Time courses of the generated H_2O_2 concentrations. (b) H_2O_2 production rates. The error bars represent one standard deviation.

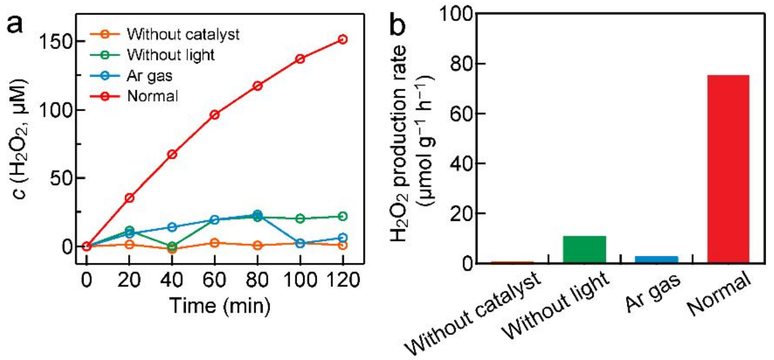

Figure S12. Photocatalytic H_2O_2 production under different conditions. The conditions of no catalyst, no light irradiation, or under Ar gas atmosphere were employed. (a) Time courses of the generated H_2O_2 concentrations. (b) H_2O_2 production rates under different conditions.

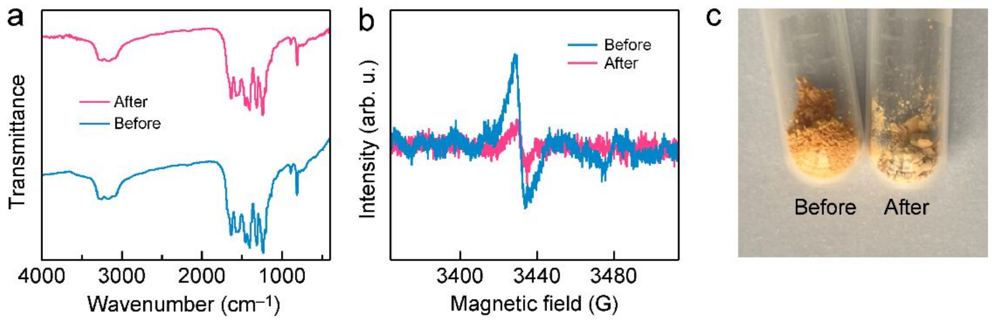

Figure S13. C_3N_4-nv photocatalyst before and after reaction. (a) FTIR spectra. (b) EPR spectra. (c) Photograph of the sample before and after reaction, showing the color change.

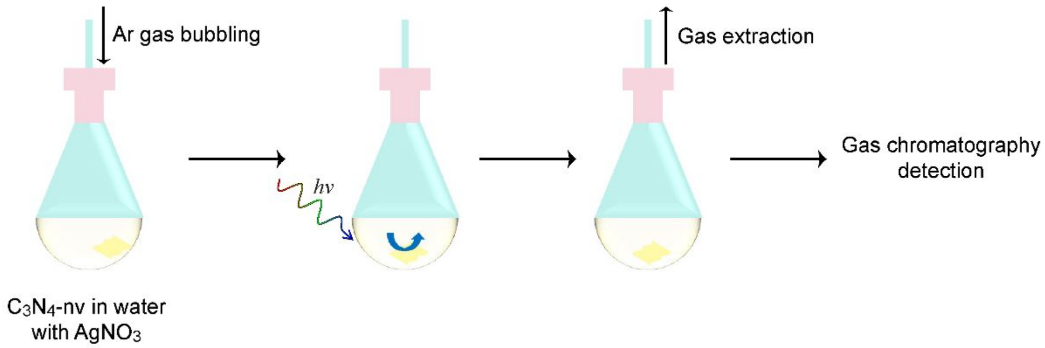

Figure S14. Schematic diagram showing the experimental procedure. It was used to confirm the oxidation product in the H_2O_2 production with the addition of $AgNO_3$ as the electron acceptor and Ar gas bubbling.

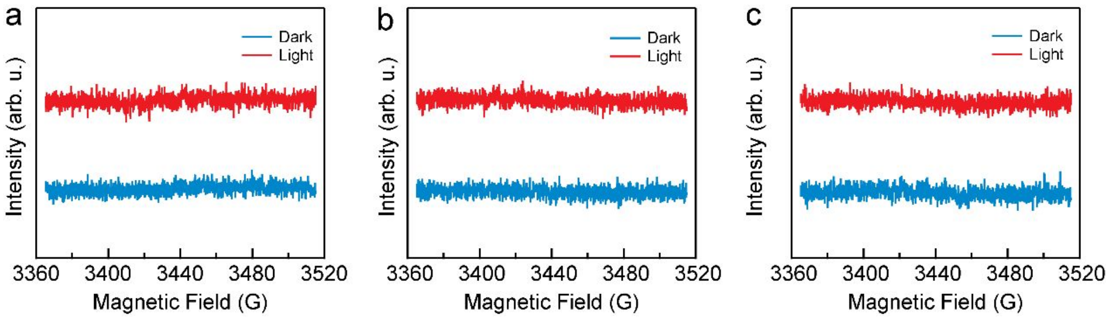

Figure S15. EPR spectra for the detection of $\bullet O_2^-$ radicals. (a) For C_3N_4-hb. (b) For C_3N_4-hb-nv. (c) For C_3N_4-nv. The samples during the detection were subjected under visible light irradiation.

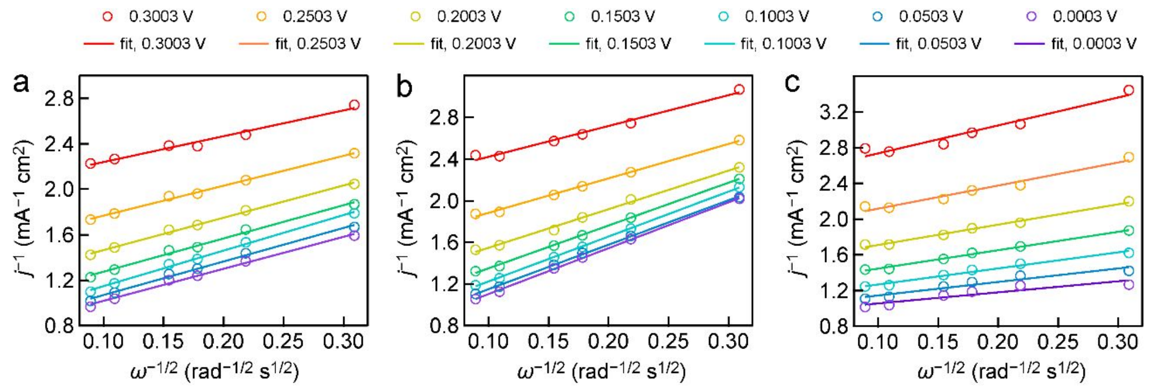

Figure S16. Koutecky-Levich plots at different potentials for the three representative photocatalyst samples. (a) C_3N_4-hb. (b) C_3N_4-hb-nv. (c) C_3N_4-nv. The samples were in the 0.1 M KOH solution saturated with O_2 gas.

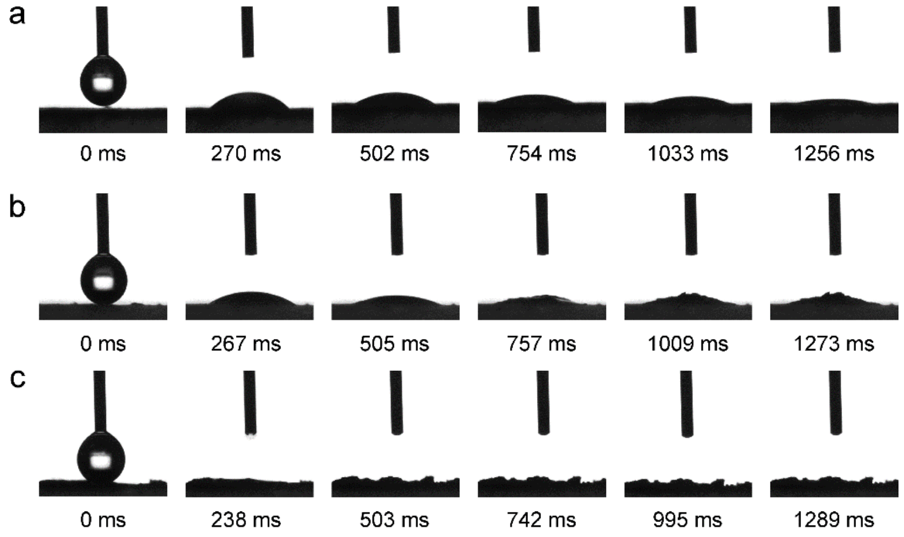

Figure S17. Contact angle images of water on the photocatalysts. (a) C_3N_4-hb. (b) C_3N_4-hb-nv. (c) C_3N_4-nv.

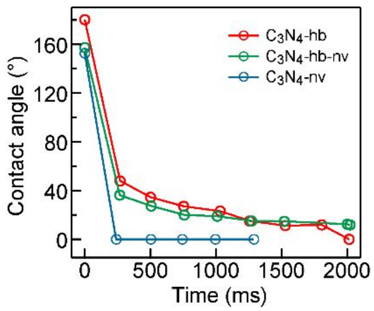

Figure S18. Contact angle variation as a function of time for C_3N_4-hb, C_3N_4-hb-nv, and C_3N_4-

nv.

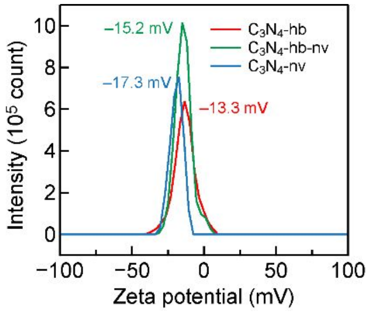

Figure S19. Zeta potentials. The C_3N_4-hb, C_3N_4-hb-nv, and C_3N_4-nv samples were dispersed in water during the measurements.

Table S1. Binding Energies of the C 1s Core Electrons of the Three Representative Samples

Sample	Element	Peak 1: C–C		Peak 2: (C)$_3$–N		Peak 3: N–C=N		Peak 1: Peak 2: Peak 3
		Position (eV)	Area	Position (eV)	Area	Position (eV)	Area	
C_3N_4-hb	C	284.828	17326.8	287.161	6751.1	288.163	57609.2	21.24%: 8.26%: 70.50%
C_3N_4-hb-nv	C	284.845	24715.8	286.940	7059.7	288.080	58895.8	27.26%: 7.79%: 64.95%
C_3N_4-nv	C	284.776	28246.0	287.440	17441.2	288.097	48342.9	30.06%: 18.55%: 51.39%

Table S2. Binding Energies of the N 1s Core Electrons of the Three Representative Samples

Sample	Element	Peak 1: C=N–C		Peak 2: N_{3C}		Peak 3: NH_x groups		Peak 1: Peak 2: Peak 3
		Position (eV)	Area	Position (eV)	Area	Position (eV)	Area	
C_3N_4-hb	N	398.619	104639.4	400.040	25546.9	401.190	10147.2	74.55%: 18.22%: 7.23%
C_3N_4-hb-nv	N	398.530	96768.5	399.987	36678.5	401.159	4788.4	70.00%: 26.53%: 3.46%
C_3N_4-nv	N	398.514	91237.5	399.989	36983.0	401.230	8525.7	66.71%: 27.06%: 6.23%

Table S3. Summary of the PL Decay Time Values and Their Relative Amplitudes for C_3N_4-hb, C_3N_4-hb-nv, and C_3N_4-nv Obtained from the Transient PL Spectra by Biexponential Decay Fitting

Sample	Delay time (ns)		Relative amplitude (%)		Average lifetime (τ, ns)
	τ_1	τ_2	f_1	f_2	
C_3N_4-hb	4.21	33.65	61.13	38.87	6.38
C_3N_4-hb-nv	7.34	51.66	45.74	54.26	13.73
C_3N_4-nv	7.22	501.52	9.84	90.16	64.79

Table S4. Determined AQEs for the Photocatalytic H_2O_2 Production at Different Wavelengths

Wavelength (nm)	H_2O_2 produced (μmol h^{-1})	Light power density I (mW cm^{-2})	Light power P (mW)	AQE (%)
420	37.116	7.6	309.472	1.90
475	52.194	10.9	443.848	1.65
520	24.180	11.3	460.136	1.01
550	27.702	10.0	407.200	0.82
600	9.960	10.7	435.704	0.25
650	4.740	11.2	456.064	0.11
700	4.254	11.5	468.280	0.09

Table S5. Comparison of Photocatalytic H_2O_2 Production Performance in Pure Water Based on Carbon Nitride in Recent Representative Works

Catalyst	Catalyst concentration (mg mL^{-1})	Irradiation	Production rate (µmol g^{-1} h^{-1})	AQE (%)	LCCE (%)	Ref.
C_3N_4-nv	1.0	λ > 420 nm	75.66	1.9% at 420 nm	3.85	This work
CNIO-GaSA	1.0	λ > 420 nm	331.7	7.1% at 459 nm	0.40	[1]
g-C_3N_4/PDI$_x$	1.7	λ > 420 nm	21.1	2.6% at 420 nm		[2]
g-C_3N_4/PDI/rGO$_x$	1.7	λ > 420 nm	24.2	6.1% at 420 nm	0.20	[3]
g-C_3N_4/MTI	1.7	λ > 420 nm	22.9	6.1% at 420 nm	0.18	[4]
g-C_3N_4/PDI-BN-rGO	1.7	λ > 420 nm	30.8	7.3% at 420 nm	0.27	[5]
Sb-SAPC15	2.0	λ > 420 nm	58.8	17.6% at 420 nm	0.61	[6]
g-C_3N_4-PWO	1.0	λ > 420 nm	63.0			[7]
OCN-500	1.0	λ > 420 nm	106.0	10.2% at 420 nm		[8]
PEI/C_3N_4	1.0	Simulated AM1.5G sunlight	208.1	2.12% at 420 nm	0.045	[9]

Table S6. Calculated k_f and k_d Values in the H_2O_2 Photodecomposition Process

Catalyst	$[H_2O_2]$ (μM min^{-1})	k_d (min^{-1})	k_f (μM min^{-1})
C_3N_4-hb	0.030	0.530	0.022
C_3N_4-hb-nv	0.250	0.541	0.188
C_3N_4-nv	1.260	0.300	1.080

REFERENCES

(1) Tan, H.; Zhou, P.; Liu, M. X.; Zhang, Q. H.; Liu, F. Y.; Guo, H. Y.; Zhou, Y.; Chen, Y.; Zeng, L. Y.; Gu, L.; Zheng, Z. F.; Tong, M. P.; Guo, S. J. Photocatalysis of water into hydrogen peroxide over an atomic Ga-N_5 site. *Nat. Synth.* **2023**, *2*, 557–563.

(2) Shiraishi, Y.; Kanazawa, S.; Kofuji, Y.; Sakamoto, H.; Ichikawa, S.; Tanaka, S.; Hirai, T. Sunlight-driven hydrogen peroxide production from water and molecular oxygen by metal-free photocatalysts. *Angew. Chem. Int. Ed.* **2014,** *53*, 13454–13459.

(3) Kofuji, Y.; Isobe, Y.; Shiraishi, Y.; Sakamoto, H.; Tanaka, S.; Ichikawa, S.; Hirai, T. Carbon nitride–aromatic diimide–graphene nanohybrids: metal-free photocatalysts for solar-to-hydrogen peroxide energy conversion with 0.2% efficiency. *J. Am. Chem. Soc.* **2016**, *138*, 10019–10025.

(4) Kofuji, Y.; Ohkita, S.; Shiraishi, Y.; Sakamoto, H.; Ichikawa, S.; Tanaka, S.; Hirai, T. Mellitic triimide-doped carbon nitride as sunlight-driven photocatalysts for hydrogen peroxide production. *ACS Sustainable Chem. Eng.* **2017**, *5*, 6478–6485.

(5) Kofuji, Y.; Isobe, Y.; Shiraishi, Y.; Sakamoto, H.; Ichikawa, S.; Tanaka, S.; Hirai, T. Hydrogen peroxide production on a carbon nitride–boron nitride-reduced graphene oxide hybrid photocatalyst under visible light. *ChemCatChem* **2018**, *10*, 2070–2077.

(6) Teng, Z. Y.; Zhang, Q. T.; Yang, H. B.; Kato, K.; Yang, W. J.; Lu, Y.-R.; Liu, S. X.; Wang, C. Y.; Yamakata, A.; Su, C. L.; Liu, B.; Ohno, T. Atomically dispersed antimony on carbon nitride for the artificial photosynthesis of hydrogen peroxide. *Nat. Catal.* **2021**, *4*, 374–384.

(7) Zhao, S.; Zhao, X. Polyoxometalates-derived metal oxides incorporated into graphitic

carbon nitride framework for photocatalytic hydrogen peroxide production under visible light. *J. Catal.* **2018**, *366*, 98–106.

(8) Wei, Z.; Liu, M. L.; Zhang, Z. J.; Yao, W. Q.; Tan, H. W.; Zhu, Y. F. Efficient visible-light-driven selective oxygen reduction to hydrogen peroxide by oxygen-enriched graphitic carbon nitride polymers. *Energy Environ. Sci.* **2018**, *11*, 2581–2589.

(9) Zeng, X. K.; Liu, Y.; Kang, Y.; Li, Q. Y.; Xia, Y.; Zhu, Y. L.; Hou, H. L.; Uddin, M. H.; Gengenbach, T. R.; Xia, D. H.; Sun, C. H.; Mccarthy, D. T.; Deletic, A.; Yu, J. G.; Zhang, X. W. Simultaneously tuning charge separation and oxygen reduction pathway on graphitic carbon nitride by polyethylenimine for boosted photocatalytic hydrogen peroxide production. *ACS Catal.* **2020**, *10*, 3697–3706.
